# Ras GTPase-Like Protein MglA, a Controller of Bacterial Social-Motility in Myxobacteria, Has Evolved to Control Bacterial Predation by *Bdellovibrio*

**DOI:** 10.1371/journal.pgen.1004253

**Published:** 2014-04-10

**Authors:** David S. Milner, Rob Till, Ian Cadby, Andrew L. Lovering, Sarah M. Basford, Emma B. Saxon, Susan Liddell, Laura E. Williams, R. Elizabeth Sockett

**Affiliations:** 1 Centre for Genetics and Genomics, School of Life Sciences, University of Nottingham, Medical School, Nottingham, United Kingdom; 2 School of Biosciences, University of Birmingham, Birmingham, United Kingdom; 3 School of Biosciences, University of Nottingham, Sutton Bonington, Nottinghamshire, United Kingdom; 4 Bacterial Epidemiology and Antimicrobial Resistance Research Unit, Agricultural Research Service, United States Department of Agriculture, Athens, Georgia, United States of America; VIB and KULeuven, Belgium

## Abstract

*Bdellovibrio bacteriovorus* invade Gram-negative bacteria in a predatory process requiring Type IV pili (T4P) at a single invasive pole, and also glide on surfaces to locate prey. Ras-like G-protein MglA, working with MglB and RomR in the deltaproteobacterium *Myxococcus xanthus*, regulates adventurous gliding and T4P-mediated social motility at both *M. xanthus* cell poles. Our bioinformatic analyses suggested that the GTPase activating protein (GAP)-encoding gene *mglB* was lost in *Bdellovibrio*, but critical residues for MglA_Bd_ GTP-binding are conserved. Deletion of *mglA_Bd_* abolished prey-invasion, but not gliding, and reduced T4P formation. MglA_Bd_ interacted with a previously uncharacterised tetratricopeptide repeat (TPR) domain protein Bd2492, which we show localises at the single invasive pole and is required for predation. Bd2492 and RomR also interacted with cyclic-di-GMP-binding receptor CdgA, required for rapid prey-invasion. Bd2492, RomR_Bd_ and CdgA localize to the invasive pole and may facilitate MglA-docking. Bd2492 was encoded from an operon encoding a TamAB-like secretion system. The TamA protein and RomR were found, by gene deletion tests, to be essential for viability in both predatory and non-predatory modes. Control proteins, which regulate bipolar T4P-mediated social motility in swarming groups of deltaproteobacteria, have adapted in evolution to regulate the anti-social process of unipolar prey-invasion in the “lone-hunter” *Bdellovibrio*. Thus GTP-binding proteins and cyclic-di-GMP inputs combine at a regulatory hub, turning on prey-invasion and allowing invasion and killing of bacterial pathogens and consequent predatory growth of *Bdellovibrio*.

## Author Summary

Bacterial cell polarity control is important for maintaining asymmetry of polar components such as flagella and pili. *Bdellovibrio bacteriovorus* is a predatory deltaproteobacterium which attaches to, and invades, other bacteria using Type IV pili (T4P) extruded from the specialised, invasive, non-flagellar pole of the cell. It was not known how that invasive pole is specified and regulated. Here we discover that a regulatory protein-hub, including Ras-GTPase-like protein MglA and cyclic-di-GMP receptor-protein CdgA, control prey-invasion. In the deltaproteobacterium, *Myxococcus xanthus*, MglA, with MglB and RomR, was found by others to regulate switching of T4P in social ‘swarming’ surface motility by swapping the pole at which T4P are found. In contrast, in *B. bacteriovorus* MglA regulates the process of prey-invasion and RomR, which is required for surface motility regulation in *Myxococcus*, is essential for growth and viability in *Bdellovibrio*. During evolution, *B. bacteriovorus* has lost *mglB*, possibly as T4P-pole-switching is not required; pili are only required at the invasive pole. A previously unidentified tetratricopeptide repeat (TPR) protein interacts with MglA and is essential for prey-invasion. This regulatory protein hub allows prey-invasion, likely integrating cyclic-di-GMP signals, pilus assembly and TamAB secretion in *B. bacteriovorus*.

## Introduction

*Bdellovibrio bacteriovorus* is a small, predatory deltaproteobacterium which invades other Gram-negative bacteria wherein it replicates. *Bdellovibrio* can encounter their prey by fast motility, driven by rotation of a single flagellum in liquid environments [1], [2], or by slow gliding motility on solid surfaces [3], but do not show social- or S-motility a process that is shown by other deltaproteobacteria (discussed below).

In *Bdellovibrio* invasion into the prey cell periplasm requires T4P, thus pilus-minus cells are incapable of host/prey-dependent (HD) growth and must be cultivated on artificial media as HI - host/prey-independent - cells [4], [5]. In flagellate HD *Bdellovibrio* the T4P are at the non-flagellar pole and prey-invasion occurs only from that anterior pole. On surfaces a flagellum is not present and the *Bdellovibrio* glide bidirectionally. Both HD and HI *Bdellovibrio* can glide and invade prey on surfaces. Our study began by examining the genetics of surface motility control in *Bdellovibrio*. This work led us to find that proteins known for surface motility control in a second deltaproteobacterium, *Myxococcus xanthus*, have evolved to control predatory invasion of bacteria by *Bdellovibrio*.

Regulation of surface motility in the deltaproteobacterium *M. xanthus* (which is always non-flagellate), has been well characterised by pioneering work of the Søgaard-Andersen [6], Mignot [7], Zusman [8], Hartzell [9] and Kaiser [10] groups for its two types of bidirectional surface motility. These are social (S)-motility, swarming movement of streams of cells using retraction of T4P at alternate poles of the cells; and adventurous (A)-motility, characterised by the movement of individual cells on a surface. A-motility (or gliding), is thought to be powered by cell envelope-spanning motor-protein complexes, [11], [12], though the precise mechanism of movement is still being revealed [13]–[15]. In *M. xanthus*, T4P localize to one pole at a time. Occasionally, *M. xanthus* cells reverse direction; this involves a switch in the polarity of the two motility systems, including a switch in the pole at which T4P assembly occurs. Thus, *M. xanthus* cells can assemble T4P at both poles but at any one time, T4P are found only at one pole [16].

Recent data suggest that the four putative gliding motor-gene operons in the *B. bacteriovorus* HD100 genome are evolutionarily linked to those A-motility gene clusters in *Myxococcus* [17], with subtle distinctive absences and additions likely reflecting *Bdellovibrio* morphology and gliding differences.

*Bdellovibrio* exhibits A-motility on surfaces in a gliding process that does not use T4P [3]. In this gliding, A-motility, individual *Bdellovibrio* cells move bidirectionally, cells can follow each other along previous paths and reversals of individual cells and re-orientations are seen. Gliding may be a particularly important mechanism by which *Bdellovibrio* explores biofilms and locates bacteria to prey upon [3], [18]. It is critical for HD *Bdellovibrio* to be able to explore or leave solid surfaces by gliding (when its flagellum cannot operate). Unlike other non-predatory bacteria, *Bdellovibrio* HD cells cannot replicate outside prey without acquiring “HI mutations” to do so [19], [20], thus without surface motility they could be trapped and starve. *B. bacteriovorus* gliding motility is slow, with cells moving, on average, 16 µm hr^−1^ [3] compared to the 24–36 µm hr^−1^ of *Myxococcus* [21]. Both *B. bacteriovorus* and *M. xanthus* show reversals in gliding direction. In *Myxococcus*, reversals during surface motility are known, from the work of the Søgaard-Andersen and Mignot labs, to be regulated by a Ras-like GTPase, MglA, which polarises the cell during gliding [6], [7], and GTPase-activating protein (GAP) protein MglB, which activates the GTPase activity of MglA to inhibit cellular reversals [6], [7]. MglA is important for activation of both the A- and S- motility “engines” (S motility engines are T4P), at the alternating leading pole, during bidirectional movements [6], [7]. In the absence of MglA, *Myxococcus* is both A- and S- non-motile. This means that MglA in *M. xanthus*, in conjunction with interacting partner RomR, regulates the localization/pole-switching activity of both T4P and gliding engine component proteins, in this bipolar bacterium. Chemotactic signals via the Frz system control cellular reversals in *M. xanthus* [22] via the RomR response regulator; RomR receives signals from the chemosensory Frz system and this modulates MglA activity [23], [24]. Although *romR* is conserved in *Bdellovibrio*, the genes encoding the Frz apparatus are not.

*Bdellovibrio* gliding is controlled by the bacterial secondary messenger cyclic-di-GMP. A diguanylyl cyclase (*dgcA*) mutation abolishes gliding, rendering *Bdellovibrio* cells unable to glide out of a consumed prey cell bdelloplast on a surface, even 2 hours after making lytic pores in it [25].The c-di-GMP receptor CdgA (GVNEF – a degenerate GGDEF protein) was found to be present at the predatory pole of *B. bacteriovorus* and deletion of *cdgA* slowed prey-invasion significantly, showing a link between c-di-GMP signalling and predation [25].

Whilst the *B. bacteriovorus* HD100 genome encodes MglA (Bd3734; accession: NP_970444.1), it does not encode an MglB homologue [23]. This report caused us to ask how bipolar switching might be achieved during *Bdellovibrio* gliding on surfaces; and whether the non-equivalent poles of the monoflagellate *Bdellovibrio* in liquids might correlate with an alternative role for MglA_Bd_. Here we show that MglA_Bd_ is required for predatory invasion, as well as being associated with changes in gliding reversal behaviour in *B. bacteriovorus*, but is not required for gliding motility *per se*. This activity of MglA_Bd_ occurs without an MglB partner, but in a cell with a RomR_Bd_ homologue. Both of these latter proteins are important to the control of bipolar motility in Myxobacteria. However we show that RomR_Bd_ has an essential role for growth in *Bdellovibrio*. We also report a previously undescribed interacting protein partner of MglA, and show that MglA_Bd_ and RomR_Bd_ interact with this tetratricopeptide repeat protein (TPR) which is also required for predation. TPR is expressed from an operon that encodes a TamAB transport system and again TamA was essential for growth. Implications of this for predation and the onset of predatory growth upon prey-invasion are discussed.

Whilst MglA_Mx_ is involved in regulation of T4P-mediated social motility in *M. xanthus*, we show that MglA_Bd_ is involved in *Bdellovibrio* in the control of pilus extrusion for the process of T4P-mediated invasion of prey cells at the single predatory pole. We show that a complex of proteins, additional to the T4P, is required at the ‘biting’ pole to organise the prey-entry machinery.

## Results

### MglA is required for predatory invasion by *B. bacteriovorus* HD100

To investigate the role of MglA_Bd_, a deletion strategy was adopted screening for possible *Bdellovibrio* mutants in both prey/host-dependent (HD) and host-independent (HI) growth modes. All attempts to inactivate *mglA* in host/prey-dependent *B. bacteriovorus* HD100 were unsuccessful, despite screening many more cells than required to generate other *Bdellovibrio* deletion strains [26] (364 revertants obtained from second crossover events, but no deletion mutants, from three separate conjugations); suggesting that MglA_Bd_ is essential for an aspect of the predatory life cycle.

**Figure 1:**
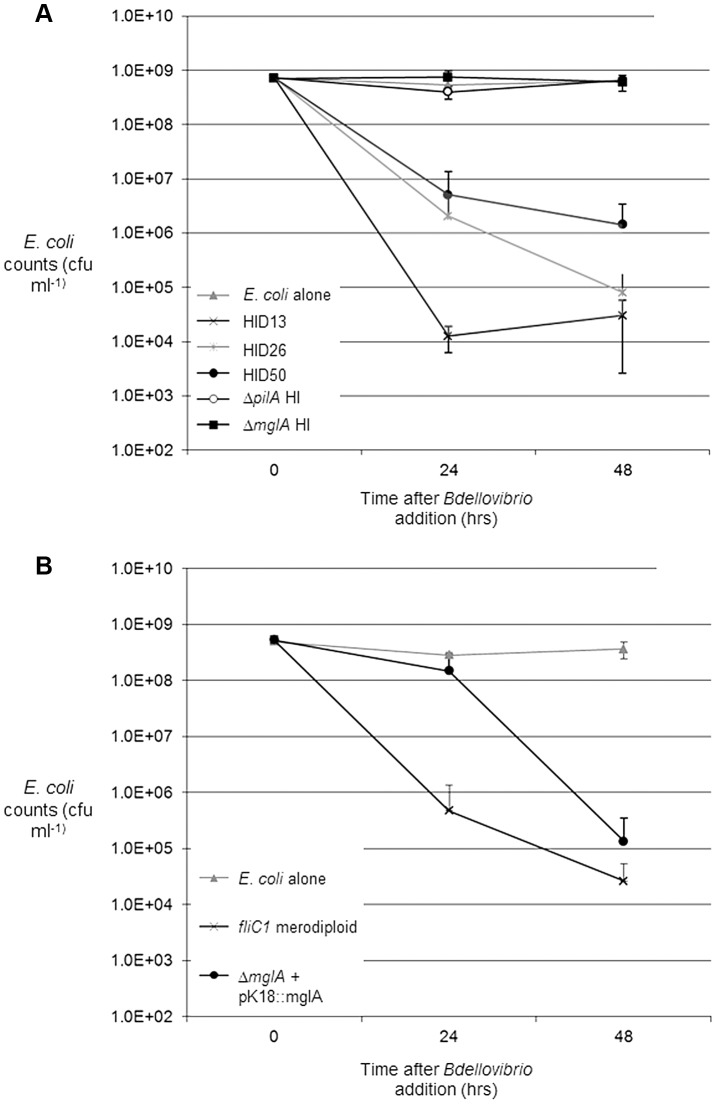
Predation and *in cis* complementation of *B. bacteriovorus *Δ*mglA* HI strains, on *E. coli* prey. (A) Predation efficiency of the Δ*mglA* HI strain was assayed against predatory and non-predatory controls by the reduction of *E. coli* numbers over 48 hours. Three wild-type HI strains (HID13, HID26 and HID50) reduced *E. coli* numbers in liquid cultures by up to four logs (grey region shows known natural variation in predation rate between different wild-type HI isolates). The Δ*mglA* HI strain showed no reduction in *E. coli* numbers, comparable to a previously-studied, non-predatory Δ*pilA* HI strain, and to *E. coli* with no added *B. bacteriovorus*. (B) Reintroduction of the *mglA* ORF *in cis* to the Δ*mglA* HI strain in plasmid pK18::*mglA* restored predatory growth. Error bars represent 1 SD from the mean (for predation-testing of **Δ** *bd2492* strain see Figure S5).

Three host-independent (HI) Δ*mglA_Bd_* strains were obtained through sucrose-suicide counter-selection from a total of 76 screened. When challenged with prey, Δ*mglA_Bd_* HI *B. bacteriovorus* strains were unable to lyse *E. coli* in either a soft agar prey-lawn on the surface of YPSC plates, or in liquid culture (Figure 1A). Introduction of wild type *mglA_Bd_* by *in cis* complementation method (as described previously [25]) restored predation (Figure 1B) confirming that MglA_Bd_ is essential for predatory growth.

**Figure 2:**
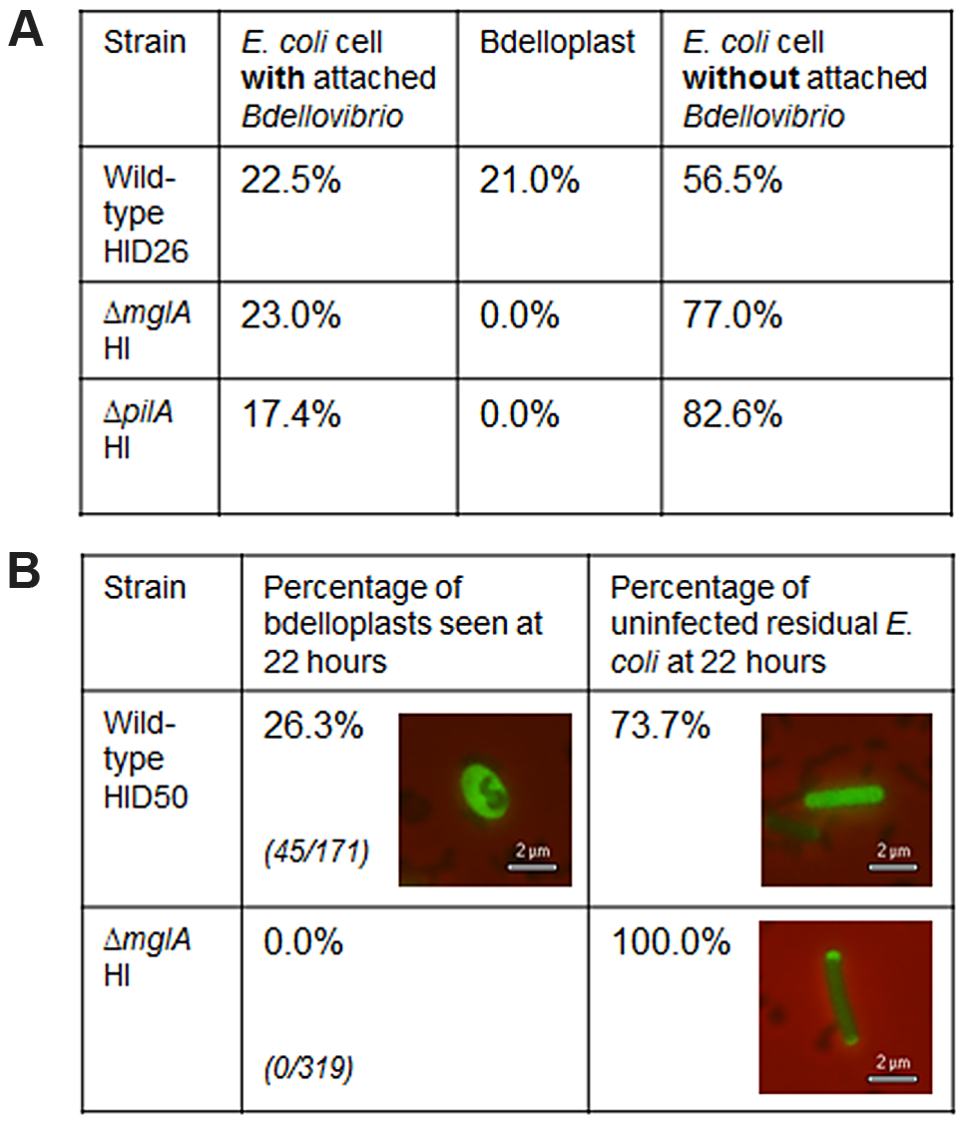
Host-independent invasion and attachment assays of Δ*mglA* strain and wild-type controls. (A) *Attachment assay*: After 1 hour, 21.0% of *E. coli* cells were attached to and invaded by wild-type *Bdellovibrio* HI strain HID26 cells. A further 22.5% of *E. coli* cells were attached to, but not invaded by HID26 cells. 23.0% and 17.4% of *E. coli* cells were attached to by Δ*mglA* HI and Δ*pilA* HI, respectively. The Δ*mglA* HI and Δ*pilA* HI strains never invaded to form bdelloplasts. The attachment assay has the following variability: Percentage points (pp): WT 43.5%±15.5 pp; Δ*mglA* 23.0%±4.2 pp; Δ*pilA* 17.4%±7.3 pp. (B) *Invasion assay*: HI wild-type *Bdellovibrio* control HID50 was able to infect *E. coli* prey cells (26.3% of *E. coli* cells invaded to form bdelloplasts after 22 hours, 45/171 *E. coli* cells). *Bdellovibrio* Δ*mglA* HI strain could not invade *E. coli* prey (0.0% of *E. coli* cells invaded to form bdelloplasts after 22 hours, 0/319 *E. coli* cells). Fluorescent images show representative fluorescent *E.coli* S17-1::pMAL_p2-mCherry cells, either uninfected or rounded to form bdelloplasts.

The Δ*mglA* HI *B. bacteriovorus* strain could not reduce *E. coli* numbers in liquid culture, though this strain could still attach to the exterior of potential prey cells (Figure 2). A parallel assay showed that 43.5% of wild-type *B. bacteriovorus* HI cells attached to, or had entered, *E. coli* prey cells after 1 hour (Figure 2A) but no Δ*mglA* HI strain formed prey-bdelloplasts (*Bdellovibrio* cause the prey to round-up into ‘bdelloplast’ structures after invasion), even after 22 hours. Figure 2 also shows that both the Δ*mglA* HI and Δ*pilA* HI (Δ*bd1290*, which is known to lack pili and is obligately host-independent [4]) could still attach to *E. coli* prey cells, albeit at a lower frequency. This suggests that pili are not a prerequisite for attachment, (although they are required for prey-invasion [4], [5]), and suggests that the Δ*mglA* HI predatory defect is not due to the inability of the *Bdellovibrio* cell to attach to prey cells.

The nature of the predatory defect of the Δ*mglA* HI strain was analysed further by microscopy, using a fluorescent *E.coli* S17-1::pMAL_p2-mCherry prey strain [27]. Addition of the Δ*mglA* HI strain to *E.coli* S17-1::pMAL_p2-mCherry and incubation for 22 hours demonstrated that although Δ*mglA* HI cells could attach to the outside of a prey cell, they could not invade to form bdelloplasts (Figure 2B). A wild-type HI *B. bacteriovorus* strain (HID50) successfully invaded *E. coli* cells and killed them (as shown in Figure 1) and at the 22 hour stage was shown to have formed bdelloplasts from 26.3% of the remaining *E. coli*, compared to zero bdelloplasts for the Δ*mglA* HI strain. Thus the deletion of *mglA_Bd_* abolished a process required for prey-invasion.

### The *B. bacteriovorus mglA* mutant is hypo-piliated

The Δ*mglA* HI strain showed a similar phenotype to that observed in a pilus-minus (Δ*pilA*) strain, which was known to be unable to invade prey cells [4]. We hypothesised that *B. bacteriovorus* Δ*mglA* might be defective in the synthesis or extrusion of pili, preventing prey cell invasion. This seemed plausible given that MglA regulates both the pole-switching of the A-motility and Type IV pilus-mediated S-motility systems in *M. xanthus*. Transmission electron microscopy of HI *Bdellovibrio* cultures grown to an OD_600_ of 0.2–0.3 showed that a wild type HI control had pili in 14.3% of cells, whilst Δ*mglA* HI had pili in only 2.3% of cells analysed (p = 0.02). These data suggested that MglA_Bd_ regulates formation of pili; loss of *mglA* reduces the number of piliated cells. But, in contrast to the Δ*pilA* strain which completely lacks pilus fibres, the total inability of Δ*mglA* cells to invade, despite the presence of a low (but significant) frequency of piliated cells, suggests that these few pili present in the Δ*mglA* cells are not competent to facilitate invasion. This could be due to a defect in pilus retraction upon attachment to prey surfaces, or a requirement for another MglA-controlled factor to mediate invasion. Candidate MglA_Bd_-interacting proteins for invasive processes are discussed later.

### MglA_Bd_ controls gliding reversal frequency but not gliding *per se*

Knowing that MglA_Bd_ controls pilus-mediated bacterial invasion in *B. bacteriovorus*, but that in *M. xanthus* both pilus-mediated S-motility and gliding A-motility are MglA controlled, we used time-lapse microscopy to observe Δ*mglA* and wild-type *B. bacteriovorus* strains for gliding motility on 1% agarose/CaHEPES. Surface motility in *B. bacteriovorus* begins after a period of incubation on an agarose surface and allows exploration of environments for potential prey.

**Figure 3:**
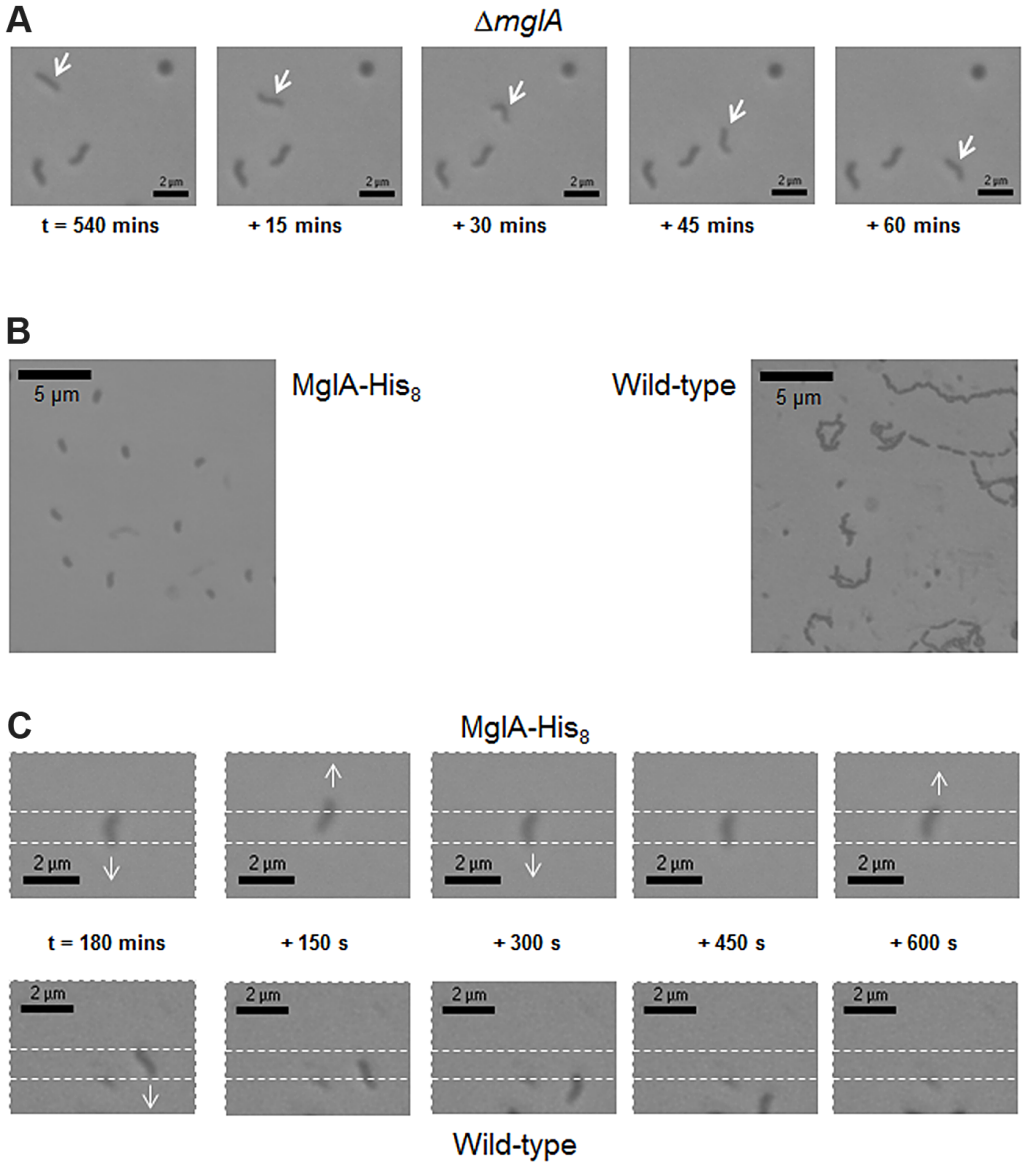
*B. bacteriovorus* Δ*mglA* HI cells show sustained gliding motility; MglA-His_8_ HD cells show hyper-reversals. (A) *B. bacteriovorus* Δ*mglA* HI cells showed gliding motility on 1% agarose/CaHEPES. Gliding was sustained and progressive (cells were not hyper-reversing), as in the arrowed cell which moved at 9.49 µm hr^−1^. Each panel is a 15 minute timepoint, starting from 540 minutes after the cells were added to the agarose surface (three “bystander” cells that have not yet commenced gliding represent a stationary marker). (B) *B. bacteriovorus* MglA-His_8_ HD cells showed a high incidence of reversals during gliding motility on 1% agarose/CaHEPES compared to wild-type cells. Each larger panel shows a “trail-montage” of 60 minutes of gliding motility (150 second per frame): MglA-His_8_ cells show no progressive gliding motility (reversing rapidly), whilst wild-type cells show sustained runs of gliding (seen as curving trails with direction changes). (C) Smaller panels show individual wild-type and MglA-His_8_ HD cells gliding from an original start point (indicated by white dashed region), starting at 180 minutes after addition to the agarose surface and at 150 second intervals; arrow indicates direction of movement.

In contrast to recent studies in *Myxococcus xanthus* which showed that a Δ*mglA_Mx_* strain is non-motile on surfaces [7], and a *mglA* ^G21V^ strain displays hyper-reversals during A-motility [6], we found that *Bdellovibrio* Δ*mglA* cells showed sustained gliding runs on surfaces (Figure 3), indicating that MglA_Bd_ is not absolutely required for *Bdellovibrio* cells to glide.

A *Bdellovibrio* strain with C-terminally His_8_-tagged MglA_Bd_, expressed from the endogenous *bd3734* promoter *in cis*, with a plasmid promoter-driven wild-type copy of *mglA_Bd_*, could be grown predatorily, in contrast to the Δ*mglA* strain which was non-predatory. In a previous study in *M. xanthus*, the presence of tagged MglA_Mx_ protein in conjunction with wild-type MglA_Mx_ allowed gliding to remain fully functional [7]. In contrast to the sustained gliding motility of the Δ*mglA_Bd_* strain (Figure 3A), the predatory *B. bacteriovorus* HD100 MglA-His_8_ showed increased reversals during gliding: on average 9.0 reversals hr^−1^ (n = 28), significantly more than wild-type HD100 cells with an average of 3.2 reversals hr^−1^ (n = 21) (p<0.001) (Figure 3B,C). The same hyper-reversal phenotype was also observed in *B. bacteriovorus* HD100 MglA-mCherry cells (data not shown).

### Differences between MglA_Bd_ and MglA_MX_ sequences may reflect diverse functions in monopolar predation versus bipolar surface motility

**Figure 4:**
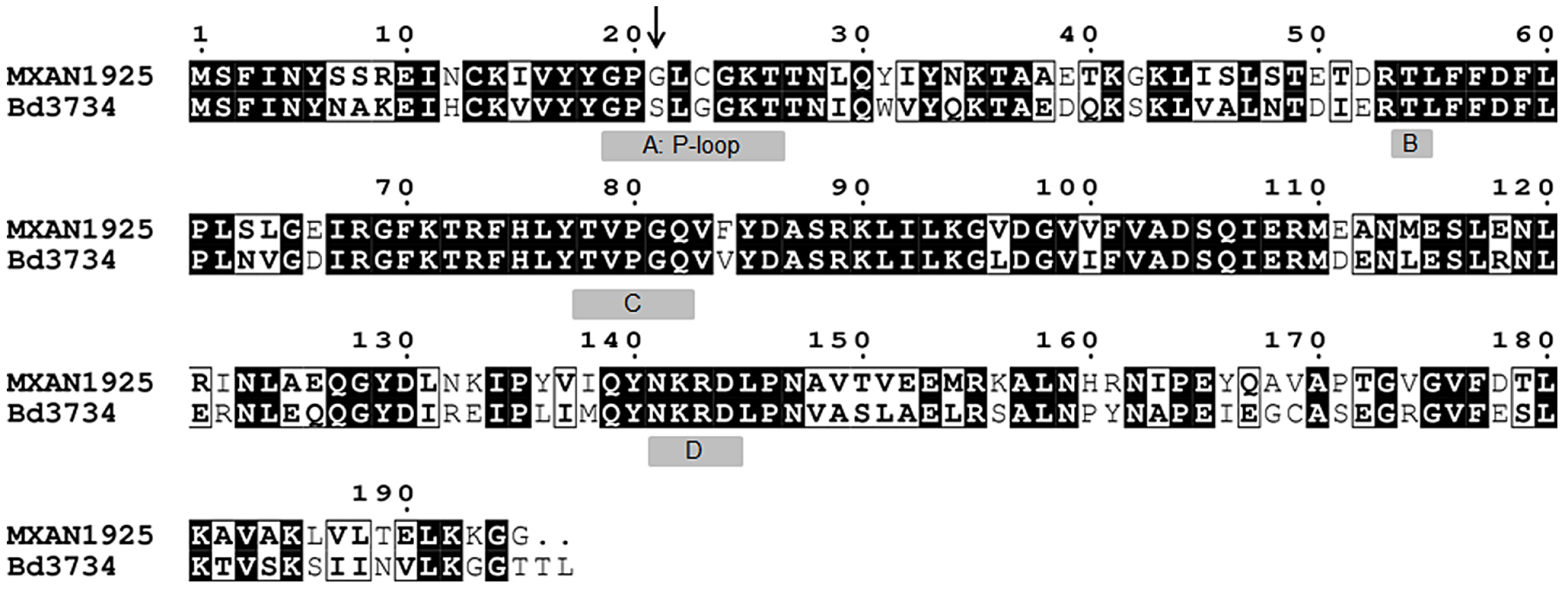
Protein alignment of MglA_Mx_ and MglA_Bd_. MglA_Mx_ (MXAN1925) alignment with MglA_Bd_ (Bd3734) shows significant sequence similarity between the two proteins. (A) The P-loop is conserved in *B. bacteriovorus*, although a serine is present in place of a glycine residue at position 21 (signified by arrow). The PM1/G1 threonine residue (B) and PM3 (C); and G2 (D) motifs are all conserved between the two proteins (for *mglA* genes, encoding MglA G21, co-occurring with *mglB* genes see Figure S1).

MglA_Bd_ (Bd3734) shares significant sequence similarity (Figure 4) with MglA_Mx_ (MXAN1925 accession: YP_630169.1), with 64% protein identity and 82% similarity (NEEDLE global alignment). The majority of residues shown to be important for MglA_Mx_ function [28], [29] are conserved in MglA_Bd_ (Figure 4A–D).

The P-loop region (_19_GXXXXGKT_26_) of MglA_Mx_ was shown by Søgaard Andersen and co-workers to be important for GTP hydrolysis, and for MglA function [28], and substitutions in this region, such as G21V, were reported to decrease hydrolysis [6]. The P-loop region of MglA_Bd_ contains a natural serine at residue 21; the corresponding G12S substitution in eukaryotic G protein Ras activates Ras protein [30], essentially locking the protein in a GTP-bound state, in the same way as a Ras G12V substitution. This suggests that MglA_Bd_ exists in a permanently GTP-bound state. The G21-equivalent residue is a conserved glycine across 7 deltaproteobacterial genera (Figure S1A) which all also have a conserved *mglB* gene, though in *Bdellovibrio* the equivalent residue is a serine.

The difference at residue 21 in the MglA_Bd_ sequence suggested to us a reason why we did not observe conservation of the gene encoding MglB in *Bdellovibrio*, as the GAP activity of an MglB would likely be ineffective on a permanently GTP-bound MglA protein such as that suggested by the MglA_Bd_ sequence with S at position 21. We thought that it might also explain the lack of a *Bdellovibrio* Frz system [23], which stimulates motility reversals in *M. xanthus*, as a mutation, causing MglA_Mx_ G21V, bypasses the requirement for Frz for reversals in that deltaproteobacterium [6]. Thus we turned to examine the presence of *mglB* in the deltaproteobacterial relatives of *Bdellovibrio*. We also tested the conserved RomR_Bd_ protein, while also looking for other proteins, specific to *Bdellovibrio*, with which MglA_Bd_ might interact. In *M. xanthus*, RomR is found at both poles of the cell and interacts with both MglA and MglB to link the Frz system to regulate polarity control [23], [24].

### *B. bacteriovorus* has lost the *mglB* gene

The majority of sequenced deltaproteobacteria genomes contain both *mglA* and *mglB*, and these are often co-transcribed at the same locus, including in *M. xanthus* where the MglB_MX_ protein has an important role in motility [6], [7], [31]. Although the *mglA* gene product in *B. bacteriovorus* HD100 shares extensive sequence similarity with other MglA proteins, there is no *mglB* homologue in the HD100 genome, despite neighbouring genes (*dnaX*, *recR*, *mglA* and a DUF149-encoding gene) showing conserved synteny to other deltaproteobacteria that do have an *mglB*. The closely related *B. bacteriovorus* Tiberius [32] also lacks an *mglB* homologue. The predatory, invasive, marine bacterium *Bacteriovorax marinus* is also closely related to *B. bacteriovorus*, although the *Bdellovibrio* and *Bacteriovorax* genera have diverged separately from Myxobacteria. A 16S rRNA phylogenetic tree of the deltaproteobacteria shows the ancestral lineage leading to *Bdellovibrio* and *Bacteriovorax* diverged from the ancestral lineage leading to the clade including *Myxococcus xanthus* [33] and in that divergent *Bdellovibrio* branch we detect *mglB* loss (Figure S1A). We found that in *B. marinus*, which also has an *mglA* gene (BMS_0054), there is an adjacent putative *mglB* homologue (BMS_0053), both genes lying downstream of *recR* (Figure S1B).

BMS_0053 shares only limited sequence similarity with other MglB Roadblock domain proteins (BMS_0053, 168 residues, shares 22% identity and 43% similarity (NEEDLE global alignment) with *M. xanthus* MglB protein, 159 residues, Figure S1C). This highly divergent MglB homologue in *Bacteriovorax* is likely still functional, since no frameshift or nonsense mutations have arisen in the *B. marinus* lineage, and protein sequence length is conserved; however, its function is unclear. We are unable to test whether *mglB* is under positive selection (dN/dS>1) in *Bacteriovorax* because synonymous substitution rates are saturated for available sequence comparisons (dS>2). The *Bacteriovorax* MglA homologue is much more conserved (66% identity and 83% similarity to MglA_Bd_) and may function in an analogous predatory role to that of *B. bacteriovorus*.

### MglA_Bd_ interacts with TPR- (tetratricopeptide repeat) domain protein Bd2492 *in vivo*

As MglA_Bd_ had both similarities and differences to MglA_Mx_, we sought to identify proteins that interact with MglA homologue Bd3734 in *B. bacteriovorus* as we reasoned that these proteins might have a predatory role. We used a pull-down co-purification assay with proteins from the predatory *B. bacteriovorus* strain producing MglA_Bd_ with a C-terminal His_8_ tag from the endogenous *mglA_Bd_* promoter, mentioned above. For the co-purification assay, a host-independent isolate of the MglA_Bd_-His_8_ strain was used, as previous array data showed that *mglA_Bd_* transcription is up-regulated in wild type HI cells, (which remain predatory but are longer than attack phase *Bdellovibrio*). Whole cell lysates of this HI strain were used in the assay, in which the bait His-tagged protein MglA_Bd_ binding to TALON-NX cobalt-charged resin allowed interacting proteins to be identified (Figure S2) that were not present in the control without the His-tag.

MglA_Bd_ co-purified with Bd2492 (accession: NP_969302.1) (Figure S2) - a *B. bacteriovorus* protein with a hypothetical annotation, with predicted tetratricopeptide repeat (TPR) domains typically involved in protein-protein interactions. Bands were excised from the gel and analysed by LC-MS/MS. Corresponding regions of the wild-type HID13 control lane were also analysed, and neither MglA_Bd_ nor Bd2492 were found in these regions, suggesting that MglA_Bd_ and Bd2492 (TPR_Bd_) interact *in vivo*.

### Confirmation that MglA_Bd_ and TPR_Bd_ proteins interact by bacterial two-hybrid and heterologous co-expression in *E. coli*

The *mglA* ORF and *bd2492* ORF were cloned into pUT18C and pKT25 vectors containing T18 and T25 fragments of adenylate cyclase, respectively [34]. The bacterial two-hybrid assay for MglA and Bd2492 showed a strong signal (Figure S3A–B) suggesting that the two *B. bacteriovorus* proteins interact. This interaction was supported by the observation that MglA co-purifies with His_6-_tagged Bd2492 in nickel-affinity chromatography of *E. coli* lysates heterologously expressing these two proteins from plasmid pD2492N/3734 (Figure S4). Gel filtration and SDS-PAGE of purified MglA and Bd2492-His_6_ indicated that the MglA-Bd2492 complex has an Mw of approximately 63 kDa and exists predominantly as a heterodimeric complex of 1∶1 stoichiometry (data not shown).

### TPR_Bd_ is required for predatory invasion by *B. bacteriovorus* HD100

As the *B. bacteriovorus mglA* mutant was non-predatory, we tested whether *bd2492* (encoding TPR_Bd_) was essential for predatory growth. All attempts to inactivate *bd2492*TPR in host-dependent *B. bacteriovorus* HD100 were unsuccessful (68 revertants obtained from second crossover events, but no deletion mutants). Two host-independent (HI) Δ*bd2492* strains were obtained through sucrose-suicide counter-selection from a total of 10 screened. When challenged with prey, Δ*bd2492*TPR HI strains were unable to lyse *E. coli* in liquid culture (Figure S5). As with the Δ*mglA* HI strains, the Δ*bd2492*TPR HI strains could still attach to *E. coli* prey cells (attachment assay; 26.6% of *E. coli* cells had attached *Bdellovibrio*), but could not invade to form bdelloplasts (invasion assay; 0/389 *E. coli* cells). The *B. bacteriovorus* Δ*bd2492*TPR HI strain was still able to glide on 1% agarose CaHEPES (data not shown).

### *B. bacteriovorus* genes *bd2492*, *bd2494* and *bd2495* are co-transcribed and syntenic in other deltaproteobacteria

**Figure 5:**
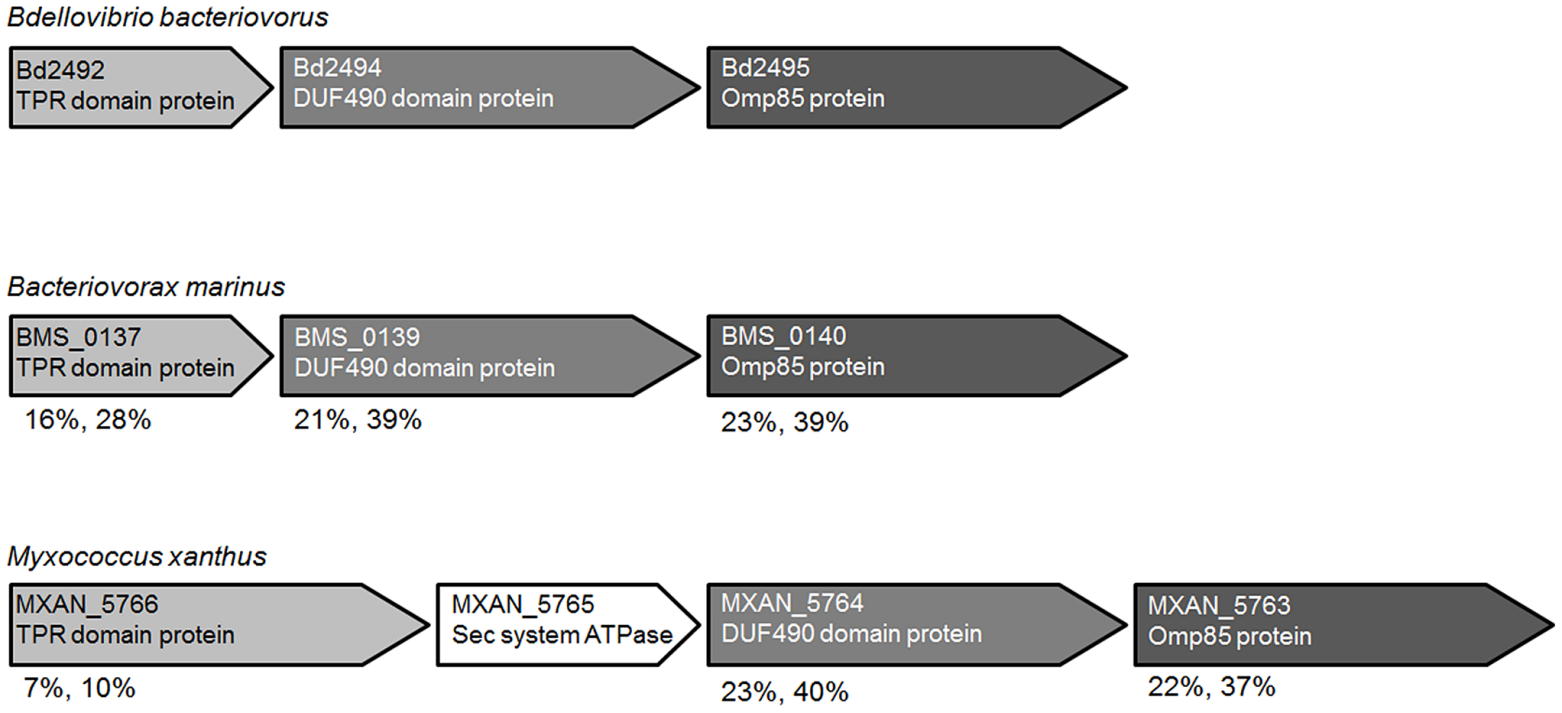
Gene synteny of *bd2492–bd2495* homologues is conserved in *B. bacteriovorus*, *M. xanthus* and *B. marinus*. Genes encoding a TPR domain protein are followed by genes encoding a DUF490 domain protein and an Omp85 superfamily protein in all three bacterial species. In *M. xanthus*, the three genes are interrupted by a gene encoding a putative Sec system ATPase, MXAN_5765. Percentage protein sequence identities and similarities with the *B. bacteriovorus* protein (NEEDLE global alignment) are shown underneath (for operon-confirmation of *bd2492-2495* see Figure S6).

TPR gene *bd2492* is co-transcribed with *bd2494* and *bd2495* (Figure S6). The same gene synteny is also found in *M. xanthus* (*MXAN*_*5763*-*5766*) and *B. marinus* SJ (*BMS*_*0137*-*140*) (Figure 5) where the gene encoding a TPR domain protein is followed by genes encoding homologues of Bd2494 and Bd2495. In *M. xanthus*, the genes encoding homologues of Bd2492 and Bd2494 (MXAN_5766 and MXAN_5764) are interrupted by a gene encoding a putative Sec system ATPase, MXAN_5765.

*B. bacteriovorus* gene *bd2492* encodes a hypothetical 353 amino acid tetratricopeptide repeat (TPR) protein; TPRpred (http://tprpred.tuebingen.mpg.de/tprpred) was used to predict TPR domains [35]. TPRpred confirmed that both BMS_0137 (524 residues; accession: YP_005034048.1) and MXAN_5766 (1031 residues; accession: YP_633903.1) are also predicted to contain TPR domains. All three TPR domain proteins do not have predicted signal sequences, as predicted by SignalP [36].

Bd2494 is a predicted transmembrane protein with a DUF490 domain. Both BMS_0139 (accession: YP_005034049.1) and MXAN_5764 (accession: YP_633901.1) also contain predicted DUF490 domains. Bd2495 is a surface antigen variable number repeat domain protein of the (outer membrane protein) Omp85 (TamA/BamA/YaeT) superfamily, hereafter termed TamA_Bd_; homologues of which are conserved in both *B. marinus* (BMS_0140; accession: YP_005034050.1) and *M. xanthus* (MXAN_5763; accession: YP_633900.1).

### RomR_Bd_ in *B. bacteriovorus* interacts with TPR_Bd_ by BTH and both proteins are located at the prey-invasion pole

As mentioned in the introduction, RomR_Mx_ interacts with the MglA_MX_ signalling system to regulate surface motility in response to Frz system signals [23], [24], but the Frz system is not conserved in *Bdellovibrio*. We assessed the interaction of the RomR_Bd_ (Bd2761; accession: NP_969553.1) with the MglA-interacting protein TPR_Bd_ (Bd2492) by bacterial two-hybrid (Figure S3A). RomR_Bd_ shares homology with the REC domain and C-terminal region of RomR_Mx_, whilst the remainder of the protein is less well conserved (Figure S7). RomR_Bd_ and TPR_Bd_ interact in the BTH assay (Figure S3A, C). We found that RomR_Bd_ and MglA_Bd_ interacted weakly, but not significantly (p = 0.18) (Figure S3B–C).

**Figure 6:**
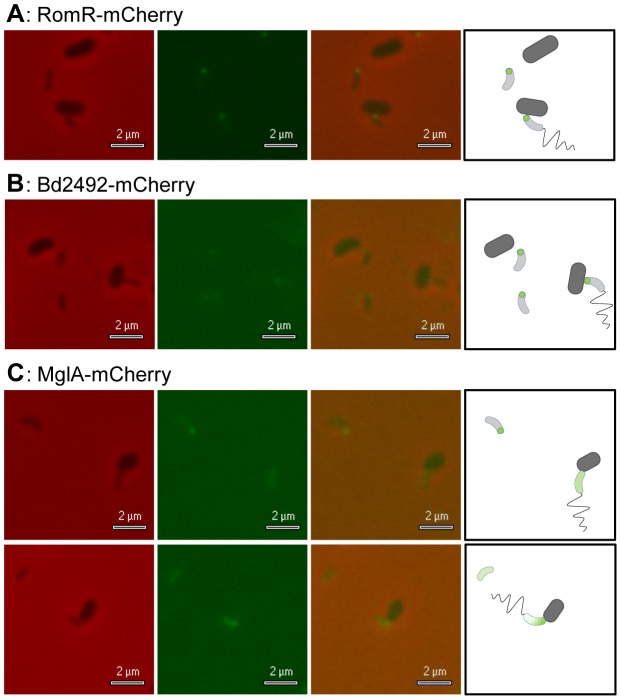
*B. bacteriovorus* RomR-mCherry and Bd2492-mCherry localised at the prey-interaction pole; MglA-mCherry showed variable diffuse foci. *B. bacteriovorus* cells were incubated with E. coli S17-1 prey cells for 5 minutes, allowing sufficient time for some of the *Bdellovibrio* cells to attach to prey. Panels- **A**: The lower prey-cell shows a typical attached *Bdellovibrio* cell, with a RomR-mCherry focus at the anterior (attached) pole of the *Bdellovibrio*. **B**: The rightmost prey-cell shows a typical attached *Bdellovibrio* cell, with a Bd2492-mCherry focus at the anterior (attached) pole of the *Bdellovibrio*. **C**: MglA-mCherry *Bdellovibrio* cells had variable foci, including diffuse and unipolar localisations. From left to right, all panels show brightfield, fluorescent, and merged images and a graphical representation. Fluorescent exposure = 2 seconds.

Fluorescent tagging of RomR_Bd_ and TPR_Bd_ with C-terminal mCherry revealed that both proteins are localised at only one pole of the cell. Co-incubation with *E. coli* prey cells confirmed that both RomR_Bd_-mCherry and TPR_Bd_-mCherry are found at the anterior, prey-interaction pole of *B. bacteriovorus* cells (Figure 6). Fluorescent tagging of MglA_Bd_ with mCherry typically showed cells with diffuse fluorescence localization in cells directly after applying to 1% agarose/CaHEPES (i.e. not gliding) (Figure 6); 63% of HD100 MglA-mCherry *Bdellovibrio* had diffuse fluorescence, the remainder showing a unipolar focus (28.4%) or bipolar foci (8.6%).

### RomR_Bd_ and TPR_Bd_ interact with invasion-pole protein CdgA, a degenerate GVNEF c-di-GMP binding protein which is required for rapid prey-invasion by *B. bacteriovorus*

We found earlier that the *Bdellovibrio* Δ*mglA* strain does not show a hyper-reversal or non-motility phenotype (Figure 3). Thus, the regulation of MglA_Bd_ localization in the control of gliding reversals (in the absence of MglB and Frz) is likely to employ an alternative signalling system to that of *M. xanthus*. Previous work suggested that this could be c-di-GMP as we have shown [25] that lack of GGDEF protein Bd0367 DgcA abolished gliding exit from bdelloplasts.

We had also had previously noted a link between a c-di-GMP binding protein and prey-invasion in *Bdellovibrio* [25]. Degenerate GGDEF (GVNEF) protein CdgA, Bd3125 (accession: NP_969891.1), is located at the prey invading pole of *B. bacteriovorus* and lack of this polar protein causes a very significant slowing of prey-invasion with bdelloplast formation taking 40–90 minutes compared to 30–40 minutes for wild type [25]. We concluded in that paper that “CdgA organises processes at the *Bdellovibrio* “nose” that are crucial to rapid prey-invasion”. In our current study, we found that both RomR_Bd_ and TPR_Bd_ (though not MglA) interacted with CdgA in the bacterial two-hybrid assay (Figure S3), supporting this idea. Whether RomR_Bd_ has a role in the regulation of gliding motility will be the subject of a subsequent study, but our interaction data suggested a link between RomR_Bd_ and predatory growth (as Δ*cdgA* was affected in predation [25]), so we tested for a *romR_Bd_* deletion strain.

### RomR_Bd_ is essential in *B. bacteriovorus*

Given the CdgA and TPR_Bd_ interactions found at the *B. bacteriovorus* invasive pole, we speculated that RomR_Bd_ would be required for prey-invasion. Attempts to delete *romR_Bd_*, both predatorily (HD) and host-independently (HI), were unsuccessful (HD 104; HI 120 revertants screened), suggesting that RomR_Bd_ is required for **both** predatory and host-independent *Bdellovibrio* growth.

### TamA_Bd_, encoded from the operon encoding TPR_Bd_ is also essential in *B. bacteriovorus*

As RomR_Bd_ interacted, by BTH, with TPR_Bd_, encoded in an operon with the *tamAB* genes, we speculated that the TamAB complex would also be required for predatory growth. Attempts to delete *tamA_Bd_* also proved unsuccessful (HD 140; HI 97 revertants screened), suggesting that TamA_Bd_ is also essential for both phases of *Bdellovibrio* growth.

## Discussion

Here we report that *B. bacteriovorus* use homologues of adventurous/social motility-control proteins for the process of predatory invasion of other bacteria. Whilst non-invasive *M. xanthus* utilise the proteins MglA and MglB to control bipolar, bidirectional surface motility [6], [7] in *Bdellovibrio* MglA_Bd_ has evolved to function without an MglB homologue (the *mglB* gene is absent) to regulate prey entry at a single pole.

### A unipolar role for the *B. bacteriovorus* homologues of the *M. xanthus* motility proteins

There are three lines of evidence to suggest this: (1) The deletion of *mglA_Bd_* caused a non-prey-invasive phenotype (Figure 1) and severely reduced pilus formation on the cell surface; (2) the natural substitution in MglA_Bd_ of serine for glycine (Figure 4) at the position equivalent to residue 21 in MglA_Mx_ suggests that MglA_Bd_ exists in a permanently GTP-bound state, and is not involved in the GTPase cycle which is key to the alternate bi-polar switching of motility proteins in *M. xanthus* [6], [7]; (3) RomR-mCherry is unipolar in *B. bacteriovorus* (Figure 6), in contrast to its asymmetric bipolar localization in *Myxococcus*, controlling MglA_Mx_ positioning.

### RomR_Bd_ is localised only at the prey-invasion pole and has a different phenotype to RomR_Mx_

We had hypothesised that RomR_Bd_ might be involved in regulating pole activity to control gliding motility. As RomR_Bd_ was found at the predatory pole only, this suggested an alternative role. We could not detect a significant interaction between RomR_Bd_ and MglA_Bd_ by BTH, but we did detect a significant interaction with Bd2492 TPR protein (Figure S3), which is also at the anterior pole (discussed later).

The RomR_Bd_ location at the anterior pole of *B. bacteriovorus* puts it where prey-invading T4P are located. Lotte Søgaard-Andersen's group showed that an *mglA*_Mx_ deletion mutant resulted in unipolar RomR_Mx_, with RomR_Mx_ and T4P, (used in that bacterium for bipolar social motility), found at the same pole [37]. Sequence- and localization- differences between unipolar RomR_Bd_ and MglA_Bd_ (in the absence of an MglB) in *B. bacteriovorus*, versus those in *M. xanthus* (which has MglB), might explain why T4P are only found at the anterior *Bdellovibrio* pole where they control prey-invasion.

Deletion of *romR_Bd_* abolished *Bdellovibrio* growth in both HI and predatory conditions, but in *M. xanthus romR* is viable with abolition of gliding motility and reduction of T4P-dependent social motility [23], [24]. Thus RomR_Bd_, which does show some sequence divergence from RomR_Mx_ (Figure S7), could be reporting T4P activity and prey-invasion, at the anterior pole, back to initiate *Bdellovibrio* growth. It should be recalled that predatory “attack phase” *Bdellovibrio* do not replicate outside prey, but initiate replication when prey are entered [20].

### Absence of MglB in *Bdellovibrio* is consistent with unipolar RomR_Bd_

Our BTH interaction data were too weak to prove a significant interaction between RomR_Bd_ and MglA_Bd_. This could be interpreted to mean that RomR_Bd_ transiently docks with MglA_Bd_ when RomR_Bd_ is complexed at the pole, that other partner proteins are required to contribute to this interaction, or that they do not interact, in contrast to published data for MglA_Mx_ [23], [24]. Our finding that RomR_Bd_ is unipolar fits with evidence in *M. xanthus* that MglB_MX_ is required for bipolar localization of RomR_Mx_ [23] and the apparent loss of MglB from the prey-invasive *Bdellovibrio* lineage in evolution. The *Bdellovibrio*-like invasive *B. marinus* has a putative *mglB* gene, the product of which shows only limited sequence similarity to other MglB Roadblock domain proteins (Figure S1). This *mglB_Bm_* gene is highly divergent from *mglb_Mx_* but likely still functional. It may be undergoing selection to evolve an alternative function, while the *B. marinus mglA* gene is maintained for a predatory role analogous to that in *B. bacteriovorus*.

MglA and MglB were shown to be conserved by Keilberg and co-workers in many deltaproteobacteria but also occur in some evolutionarily distant bacteria such as the green non sulphur bacteria, Acidobacteria and Deinococcus-Thermus group, Figure S3 in Ref [23]. The authors calculated the following: out of a total of 70 species with (at least one) predicted MglA homologue 87% = 61/70 species have MglB and an MglA. Of the 9 without MglB, 4 bacteria had MglA G21 with no MglB; 5 had MglA A/S21 with no MglB. Of these 9 with no MglB, only *B. bacteriovorus* and one other species, (a soil Acidobacterium named *Candidatus koribacteria versatilis*), have predicted RomR homologues. Thus bacteria with RomR and MglA and B may have interacting protein complexes that move between poles; but our study on *B. bacteriovorus* is the first to examine the situation in a bacterium where MglA and RomR are present but MglB is not.

As mentioned above, we detected an interaction with an additional protein that could contribute to the localization of MglA_Bd_ and RomR_Bd_ at the single prey-invasion pole of *Bdellovibrio*. This was with the unipolar tetratricopeptide repeat TPR protein, Bd2492 (TPR_Bd_) shown using both His-tag pull-downs and BTH for MglA and BTH for RomR. TPR_Bd_ could sequester either MglA_Bd_ or RomR_Bd_ at the prey-invasive pole, regulating their freedom to interact with each other, or promoting an interaction on the TPR_Bd_ surface. Deletion of *bd2492TPR_Bd_* abolished prey-invasion in the same manner as Δ*mglA_Bd_* (Figure S5, Figure 1).

It was not possible to monitor localization of fluorescently tagged proteins informatively in the HI derivative strains of the non-predatory Δ*mglA_Bd_* and Δ*bd2492* mutants. This is because HI derivatives have pleomorphic cell morphotypes (HI cells naturally differ greatly in length and shape) [38], and indeed some long HI cells are predatory at both poles [25].

### TPR gene *bd2492* is in an operon with *tamAB* genes

The *bd2492* gene is located upstream of, and is co-expressed (Figure 5, Figure S6) in an operon with, gene *bd2494*, which encodes a transmembrane protein with a C-terminal DUF490 domain, homologous to the TamB component of the TamAB autotransporter-secretion system [39]. Bd2494 might dock with TPR_Bd_ at the prey-invasive nose. The last gene in the operon (*bd2495*) encodes a 7-POTRA (polypeptide-transport-associated)-domain, outer membrane protein (OMP) member of the Omp85 superfamily. The Omp85 protein family includes the BamA component of the BAM complex, known to receive and assemble beta barrel proteins during outer membrane growth [40]. The family also includes the TamA component of the TamAB complex, which aids autotransporter secretion [39]; and two-protein secretion (TPS) proteins [41].

The TamA and TamB genes are typically adjacent in proteobacteria [39], suggesting that the adjacent *B. bacteriovorus bd2494-2495* genes encode a TamAB-like transporter.

Thus our finding that MglA_Bd_ and RomR_Bd_ interact with a TPR protein (Figure S3), encoded from the 5′ gene of a *tamAB*-like operon, suggests that the Bd2494-2495 TamAB-like transport activity might be required for OMP/autotransporter proteins involved in predation. This may account for our observation that some pili are present on the Δ*mglA* mutant but that despite this, it does not invade due to an effect on TamAB-dependent predatory protein transport. Similarly, the Δ*bd2492* mutant was also non-predatory (Figure S5), but attached to prey. This suggests that either: TPR_Bd_ and MglA_Bd_ are important in the positioning of proteins (probably Bd2494-5 TamAB_Bd_) at the predatory pole of the *B. bacteriovorus* cell to facilitate prey entry; or that binding of RomR_Bd_ and MglA_Bd_ to TPR_Bd_ affects its activity, and that of the TamAB_Bd_ complex, regulating predatory protein secretion.

Reinforcing our observation (mentioned above) that RomR_Bd_ is essential, we found, by attempting to delete *bd2495*, that TamA_Bd_ was also essential for both HD and HI growth of *Bdellovibrio*. This suggests that the activity of the TamAB complex (possibly involving a TPR-mediated interaction with RomR_Bd_) is required for secretion of proteins required for prey-invasion and both predatory and HI growth. Potential candidates for TamAB export are proteins involved in the synthesis/secretion/maturation of extracellular polysaccharide (EPS) or polyelectrolytes; an earlier study proposed that RomR was responsible for stimulating polyelectrolyte secretion in *M. xanthus* [37]. We cannot yet define whether RomR_Bd_ activates a TamAB dependent process that is essential for predatory and HI growth, or whether it reports on the activity of a TamAB complex, via its interaction with TPR_Bd_, to regulate *Bdellovibrio* growth. This will be the subject of a further extensive genetic study.

### Conserved synteny of the *TPR tamAB* genes in *Myxococcus*

Although a TPR protein interaction with MglA_Mx_ or RomR_Mx_ has not been previously reported, the *MXAN_5766* gene encoding a TPR domain protein, from a gene cluster with similar synteny to the *B. bacteriovorus bd2492*-*2495* genes (Figure 5), has previously been implicated in *M. xanthus* S-motility by transposon studies carried out by the Hartzell group [42]. The low percentages of TPR ORF similarity/identity between MXAN_5766 and Bd2492 could reflect the greatly different protein sizes and may indicate interactions with additional protein partners in *M. xanthus*. However, in *M. xanthus*, similar TPR interactions with RomR, MglA and TamAB-like proteins could play a role in bipolar motility control. Whether or not this is the case in *M. xanthus*, it is clear that TPR, and likely TamAB_Bd_, proteins play an important role in defining the single, active, predatory pole of *Bdellovibrio*.

### A protein hub controlling predatory invasion

**Figure 7:**
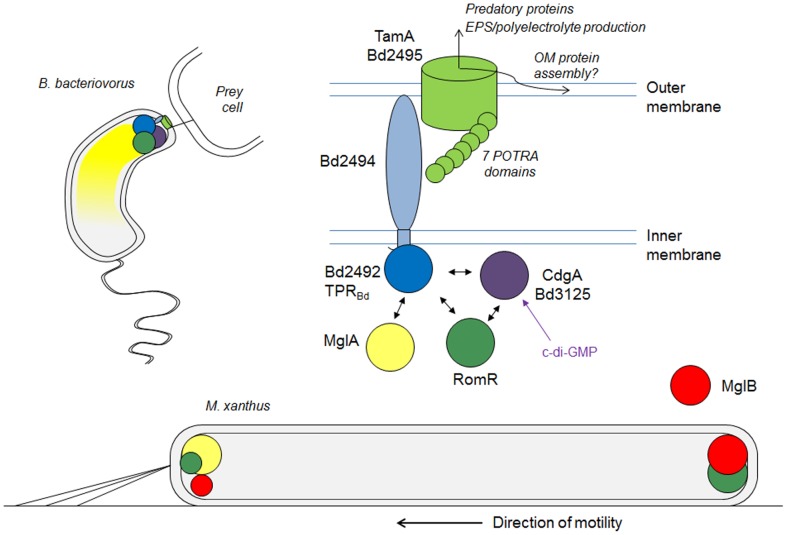
Model for *B. bacteriovorus* predatory-pole regulation during prey-invasion and its relationship to *M. xanthus* bipolar motility-control proteins. During prey-invasion; TamA_Bd_, RomR_Bd_ and CdgA protein interactions occur (see Figure S2, S3 S4) at the single *B. bacteriovorus* pole. This could control localization of the TamA_Bd_-like OMP at the prey-interaction pole or activate it to receive, (via its POTRA domains), and secrete predatory outer membrane or autotransporter proteins. The action of this secretion via Bd2495 TamA is essential to both predatory and HI lifestyles, and RomR_Bd_, (which is also essential), may regulate or report the activity of the TamAB transport system, at the single predatory pole. Additional regulation of this activity could be influenced by c-di-GMP for which CdgA, (another hub protein that binds RomR and TPR Bd2492), is a receptor in *Bdellovibrio*. MglA_Bd_ interacts with Bd2492 at the predatory pole but also is found more diffusely in the cell. MglA interactions may regulate prey entry via TPR Bd2492, as deletion of MglA or TPR Bd2492 abolishes prey-invasion but not prey attachment. MglA deletion in *Bdellovibrio* greatly reduces the level of Type IV pilus formation at the single anterior pole. In contrast Keilberg and co-workers showed that *M. xanthus* RomR and MglB localise bipolarly asymmetrically, while MglA typically localises at the leading cell pole, during surface movement, to regulate both A- and S- motility (*M. xanthus* after [23]).

We propose a predatory regulatory ‘hub’ of proteins at the *B. bacteriovorus* prey-invasive pole (Figure 7), with the TamAB-associated Bd2492-5 TPR_Bd_ protein complex involved in the organisation/assembly of OMPs or autotransporters at the predatory pole. This is reflective of TamAB protein functions in other bacteria (discussed in [39]). Such protein secretion could facilitate predation directly or produce other extra-cellular compounds such as EPS or polyelectrolytes, as mentioned above, which contribute to predatory invasion. Predatory proteins could be secreted in outer membrane vesicles (OMVs); Sar and Arf GTPases (homologous to MglA) have functions in vesicle transport [43] and *M. xanthus* vesicles likely have an extra-cellular predatory role in the “wolf-pack” [44] hunting of *M. xanthus* [45]. Our studies show that the directed prey-invasion of *Bdellovibrio* requires a protein encoded by a *tamAB* operon, suggesting synergies in TamAB-mediated predation and cell interaction processes of *B. bacteriovorus* and *M. xanthus* which is worthy of further investigation. Regulatory protein hubs are reported to control pili and flagella in other bacteria [46].

### Evolutionary comparisons in *Myxococcus* and *Bdellovibrio*

Considering evolutionary differences that led *Bdellovibrio* to prey-invasion via a single pole, we also suggest that the absence of *mglB* in *B. bacteriovorus* (and the high degree of divergence of this gene in *B. marinus*), is because MglB is no longer required for pole switching of pili: *B. bacteriovorus* pili are found at only one - the non-flagellar, prey-invasive pole [4]. This is also concordant with *B. bacteriovorus* cells being incapable of S-motility (which would require pole-switching of T4P) and instead using T4P at a single pole for prey-invasion. However, the absence of an MglB homologue does suggest that an alternative mechanism for regulating reversals during gliding motility is likely to exist.

The mechanism by which reduced incidence of pili or a change in their retraction state is caused, in the *B. bacteriovorus ΔmglA_Bd_* strain, remains to be determined. Capeness and coworkers have recently shown that regulation of *Bdellovibrio* pilus retraction status does correlate with prey-invasion [26]. Pilus retraction occurs through secretin PilQ [47], which is required for predation in *B. bacteriovorus* [18]. The OM-assembly of a pilus-biogenesis protein such as PilQ could be affected by the Bd2492-5 TamAB complex activity. Alternatively, OMPs required for secretion of EPS might be perturbed at the *Bdellovibrio* pole, preventing pilus retraction; EPS is required for pilus retraction in *M. xanthus* [48]. These considerations will be the subject of a subsequent study.

The MglA/RomR-TPR interactions reported in this paper may have evolved from ancient interactions common to ancestors of *M. xanthus* and *Bdellovibrio*, and are now used in *B. bacteriovorus* for prey-invasion control. They may also underlie the motility and “wolf-pack” predation of Myxobacteria, but the function of the *M. xanthus* TPR protein homologue remains to be explored. Pioneering work by Mignot/Theodoly has shown that adhesion during gliding motility is mediated by slime deposition [14], [15] on a solid surface and that gliding directionality is controlled by MglA_Mx_ [6], [7] and other interacting proteins. In nature gliding of *M. xanthus* may occur on top of prey bacterial biofilms and we hypothesise that the Bd2492-5 TamAB associated system may have a role in producing vesicles, not only for gliding, but to damage prey cells as part of the *M. xanthus* wolf-pack lytic process.

### Cyclic-di-GMP signalling at the predation control hub

In *M. xanthus*, chemotactic phospho-transfer signalling, involving Frz proteins, governs the localization of soluble RomR_Mx_, MglA_Mx_ and MglB_Mx_ proteins to alternately activate or deactivate each cell pole for surface-motility directionality [23], [24]. In *B. bacteriovorus*, we detected an interaction between RomR_Bd_ and the CdgA GVNEF domain c-di-GMP binding protein (Figure S3) which has been shown to affect prey entry [25]. There is no Frz system in *Bdellovibrio* [23] but our finding that CdgA binds RomR_Bd_ (Figure S3) suggests that this c-di-GMP signalling pathway could contribute to RomR_Bd_ localization in the control of the prey-invasive pole. Further work is underway to define any signalling-link to RomR_Bd_ and CdgA from our previous observations that c-di-GMP synthases control gliding motility, predation and the switch from predatory to host-independent growth [25].

The data we present here show how the “phenotype space” and function of *B. bacteriovorus* MglA has diverged from that in *M. xanthus*. MglA_Bd_ functions in the control of unipolar prey-invasion: a critical process in the predatory lifecycle of *B. bacteriovorus*. Our present observations indicate (Figure 7) that MglA_Bd_, RomR_Bd_ and the interacting TPR-domain protein TPR_Bd_ and TamAB_Bd_ complex act at a single pole in *B. bacteriovorus* to facilitate prey-invasion via a mechanism that has diverged from that which controls *M. xanthus* S-motility.

## Materials and Methods

### Bacteria, plasmids and primers

Bacterial strains and plasmids used are listed in Table S1. Primers used for gene manipulation or PCR amplification are listed in Table S2.

### Deletion construction

Markerless deletion strains of *mglA_Bd_* and *bd2492* (encoding TPR_Bd_) were generated using a modified technique of that of the Pineiro lab [49], and as described previously [25]. Construction of each mutant is described in full in Text S1.

### Fluorescent protein tagging

Fluorescent protein tags were generated as described previously [25] by cloning of a whole gene fused to mCherry at the 3′ end. Construction of each tag is described fully in Text S1.

### Fluorescent microscopy

To observe the fluorescence of *B. bacteriovorus* mCherry-tagged strains during attachment to *E. coli* prey cells, 1 ml of a *B. bacteriovorus* predatory culture (containing 2.5×10^8^ pfu ml^−1^) was concentrated 20-fold and added to a microcentrifuge tube containing 30 µl CaHEPES and 40 µl *E. coli* S17-1 pZMR100 (from a culture grown for 16 hours at 37°C 200 rpm in YT broth supplemented with Km^50^) diluted to OD_600_ 2.0 in CaHEPES, before incubating at 29°C for 5 minutes to allow attachment to occur. Cells were immobilised on a 1% agarose/CaHEPES pad and images were taken on using a Nikon Eclipse E600 epifluorescence microscope with a 100× objective lens and an hcRED filter (excitation 550 to 600 nm; emission 610 to 665 nm) with a Hamamatsu Orca ER camera. Images were analysed using Simple PCI software (version 5.3.1 Hamamatsu).

### Host-independent predation, invasion and attachment assays

Procedures for attachment, invasion and predation assays of HI *Bdellovibrio* cells on *E. coli* prey are described in Text S1. 3 biological replicates were performed.

### Gliding motility assay

*B. bacteriovorus* gliding motility was observed on 1% agarose/CaHEPES by timelapse microscopy as previously described [3]. Briefly, 1 ml of an predatory culture (containing 2.5×10^8^ pfu ml^−1^) was concentrated 10-fold (HI cultures were not concentrated) and 8 µl was spotted onto the agarose pad. Measurements of gliding reversals were calculated after cells had been gliding for >1 hr.

### Electron microscopy

To analyse percentages of piliated cells, each HI strain was back-diluted and grown to OD_600_ 0.1–0.5 in PY broth at 29°C 200 rpm. Cells were then stained with 2.0% phosphotungstic acid (PTA) on carbon formvar copper grids (Agar Scientific) and analysed for the presence/absence of a pilus structure, as described previously [26].

### Bacterial two-hybrid and protein co-purification

Procedures for bacterial two-hybrid and protein co-purification are described in Text S1.

## Supporting Information

Figure S1 Tree showing co-evolution of G21-encoding *mglA* with *mglB* versus lone *mglA* S21 in deltaproteobacteria. (A) A Maximum Likelihood phylogenetic tree of deltaproteobacteria small subunit rRNA gene sequences: the majority of these bacteria encode an MglA with a G21 residue - these also encode an MglB homologue. *Bdellovibrio bacteriovorus* and *Bacteriovorax marinus* diverge separately from these *mglB*-encoding deltaproteobacteria, including *Myxococcus xanthus*. The *B. marinus* genome encodes MglA G21 and a degenerate MglB; the *B. bacteriovorus* genome encodes MglA with an S21 residue, but no MglB homologue. Tree generated using Phylogeny.fr [50] and rooted with *Shewanella onidensis*; confidence values represent approximate likelihood-ratio (aLRT) values. (B) The *mglB*-like gene of *B. marinus* (BMS_00553) is found at the same location as the *mglB* gene in *M. xanthus* (MXAN_1926; accession: YP_630170.1) (upstream of *mglA*). (C) *B. marinus* BMS_0053 has only limited sequence similarity to *M. xanthus* MglB (MXAN_1926).
(TIF)

Figure S2 MglA co-purified with hypothetical protein Bd2492 (TPR_Bd_). SDS-PAGE on 10–20% Tris-Tricine gel with protein molecular weights (left), HID13 control (left lane) and HI MglA His_8_ (right lane). Differential bands are indicated by arrows A and B. Each differential band was excised and analysed by LC-MS/MS. The lower band (A, 22.2 kDa) was identified as Bd3734 (the protein bait) and the upper band (B, 40.5 kDa) was identified as Bd2492.
(TIF)

Figure S3 Bacterial two-hybrid shows MglA and RomR interact with Bd2492; RomR and Bd2492 interact with CdgA. A bacterial two-hybrid (BTH) assay between Bd2492 and MglA produces a positive signal on spot tests (A); the interaction between pUT18C-MglA and pKT25-Bd2492 was confirmed by beta-galactosidase assay (C). A positive result was also obtained for a BTH interaction between RomR homologue Bd2761 and Bd2492 on spot tests (A); the interaction between pUT18C-RomR pKT25-Bd2492 was confirmed by beta-galactosidase assay (C). Both RomR and Bd2492 were found to interact with CdgA (Bd3125) by BTH (A). The interactions between pKT25 Bd3125 and pUT18C-RomR or pUT18C-Bd2492 were confirmed by beta-galactosidase assay (C). When MglA and RomR interactions were assayed with tags at either end of the proteins, one combination (pUT18C-RomR and pKNT25-MglA), indicated by an asterisk (2 independent transformants) reproducibly produced a positive result on spot tests suggesting these two proteins interact (B). This interaction could not be confirmed as significant by beta-galactosidase assay, suggesting there is no interaction (as detected by BTH) between RomR and MglA. Positive control (+) = pUT18-zip and pKT25-zip and negative control (−) = pUT18C and pKT25. Error bars represent 1 SD from the mean.
(TIF)

Figure S4 Purification of the MglA-Bd2492-His_6_ complex. SDS-PAGE of fractions collected during nickel purification of the MglA-Bd2492-His_6_ complex expressed in *E. coli* cells harbouring plasmid pD2492N/3734. Soluble *E. coli* lysate (lane 1); insoluble material (lane 2); flow-through from nickel agarose column (lane 3); proteins eluted from column in the presence of 40 mM imidazole (lanes 4–6) and proteins eluted in the presence of 200 mM imidazole (lane 7). The positions of MglA and Bd2492-His_6_ on the gel are marked with arrows.
(TIF)

Figure S5 Predation of *B. bacteriovorus* Δ*bd2492* HI strains assayed against predatory wild-type controls. (A) Predation efficiency of the Δ*bd2492* HI strain was assayed against predatory and non-predatory controls by the reduction of *E. coli* numbers over 48 hours. Wild-type HI strain HID26 reduced *E. coli* numbers in liquid cultures by over four logs. The Δ*bd2492* HI strain showed no reduction in *E. coli* numbers, comparable to a known non-predatory Δ*pilA* HI strain, and to *E. coli* with no added *B. bacteriovorus*. (B) Reintroduction of the *bd2492* ORF *in cis* to the Δ*bd2492* HI strain in plasmid pK18::*bd2492* restored predatory growth. Error bars represent 1 SD from the mean Error bars represent 1 SD from the mean.
(TIF)

Figure S6 *B. bacteriovorus* genes *bd2492-2495* are co-transcribed. RT-PCR on *B. bacteriovorus* HD100 attack-phase RNA showed that *bd2492* and *bd2494* (left) are co-transcribed, as are *bd2494* and *bd2495* (right). This suggests that the three genes are all co-transcribed in the same operon. Bd = attack-phase *B. bacteriovorus* RNA; Ec = *E.coli* S17-1 RNA; (−) no template; (+) *B. bacteriovorus* genomic DNA.
(TIF)

Figure S7 ClustalW protein alignment of *M. xanthus* RomR (MXAN_4461) and *B. bacteriovorus* putative RomR homologue Bd2761. The N-terminal REC domain and the C-terminal C-domain are highly conserved between the two proteins, whilst the Pro-rich linker region of *M. xanthus* RomR (MXAN_4461; accession: YP_632632.1) is not well conserved in Bd2761. A phosphorylatable aspartic acid at residue D53 of *M. xanthus* (red arrow) is conserved between the two proteins.
(TIF)

Table S1 Plasmids and strains used in this study.
(DOCX)

Table S2 Primers used in this study.
(DOCX)

Text S1 Supplemental Materials and Methods.
(DOCX)
