# Supplementary figures and images for: Ras GTPase-Like Protein MglA, a Controller of Bacterial Social-Motility in Myxobacteria, Has Evolved to Control Bacterial Predation by Bdellovibrio
Source: PLoS Genet. 2014 Apr 10;10(4):e1004253. doi: 10.1371/journal.pgen.1004253 (PMC3983030; doi:10.1371/journal.pgen.1004253)

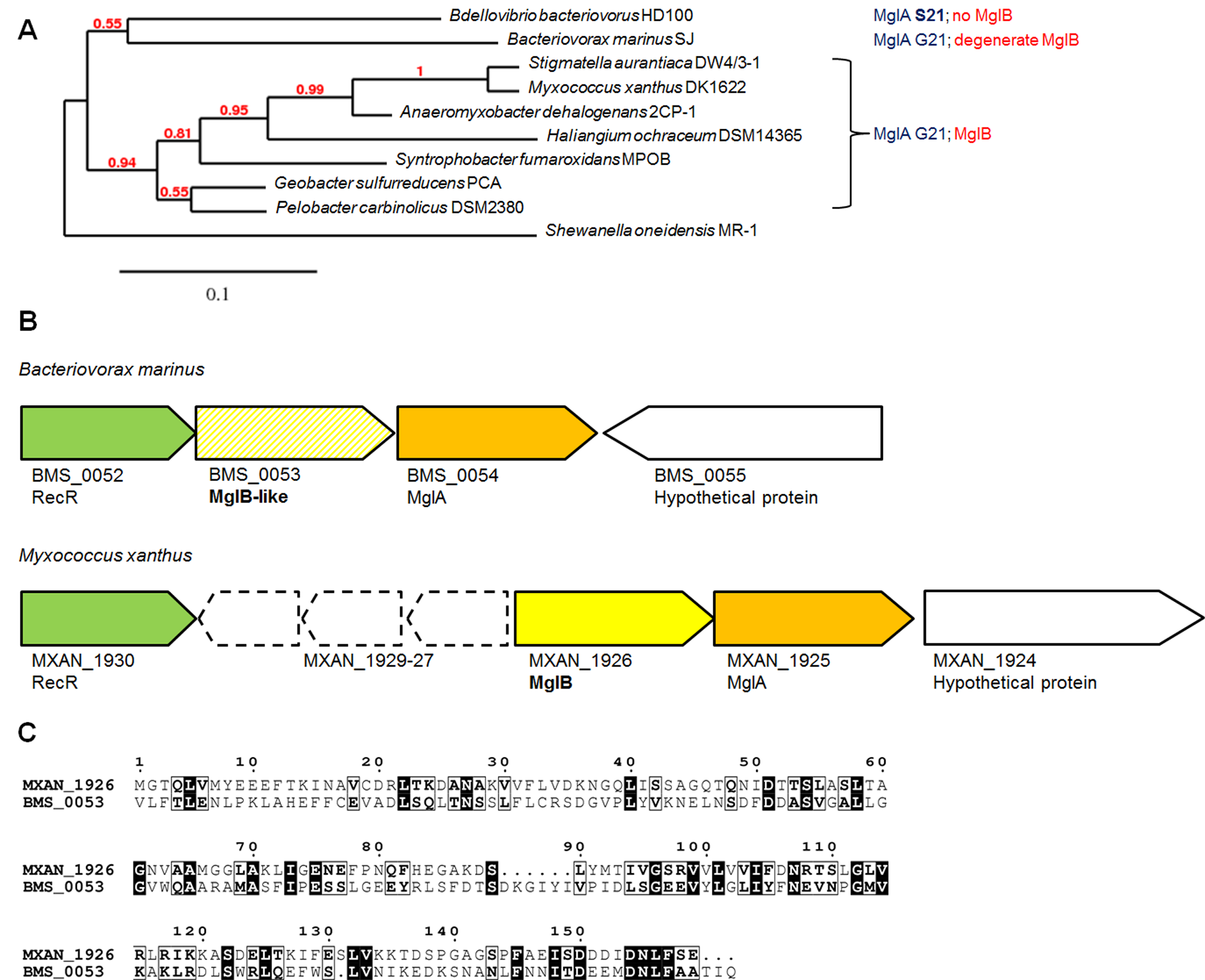

Supplement: Figure S1 — Tree showing co-evolution of G21-encoding mglA with mglB versus lone mglA S21 in deltaproteobacteria. (A) A Maximum Likelihood phylogenetic tree of deltaproteobacteria small subunit rRNA gene sequences: the majority of these bacteria encode an MglA with a G21 residue - these also encode an MglB homologue. Bdellovibrio bacteriovorus and Bacteriovorax marinus diverge separately from these mglB-encoding deltaproteobacteria, including Myxococcus xanthus. The B. marinus genome encodes MglA G21 and a degenerate MglB; the B. bacteriovorus genome encodes MglA with an S21 residue, but no MglB homologue. Tree generated using Phylogeny.fr [50] and rooted with Shewanella onidensis; confidence values represent approximate likelihood-ratio (aLRT) values. (B) The mglB-like gene of B. marinus (BMS_00553) is found at the same location as the mglB gene in M. xanthus (MXAN_1926; accession: YP_630170.1) (upstream of mglA). (C) B. marinus BMS_0053 has only limited sequence similarity to M. xanthus MglB (MXAN_1926). (TIF) [file pgen.1004253.s001.tif]

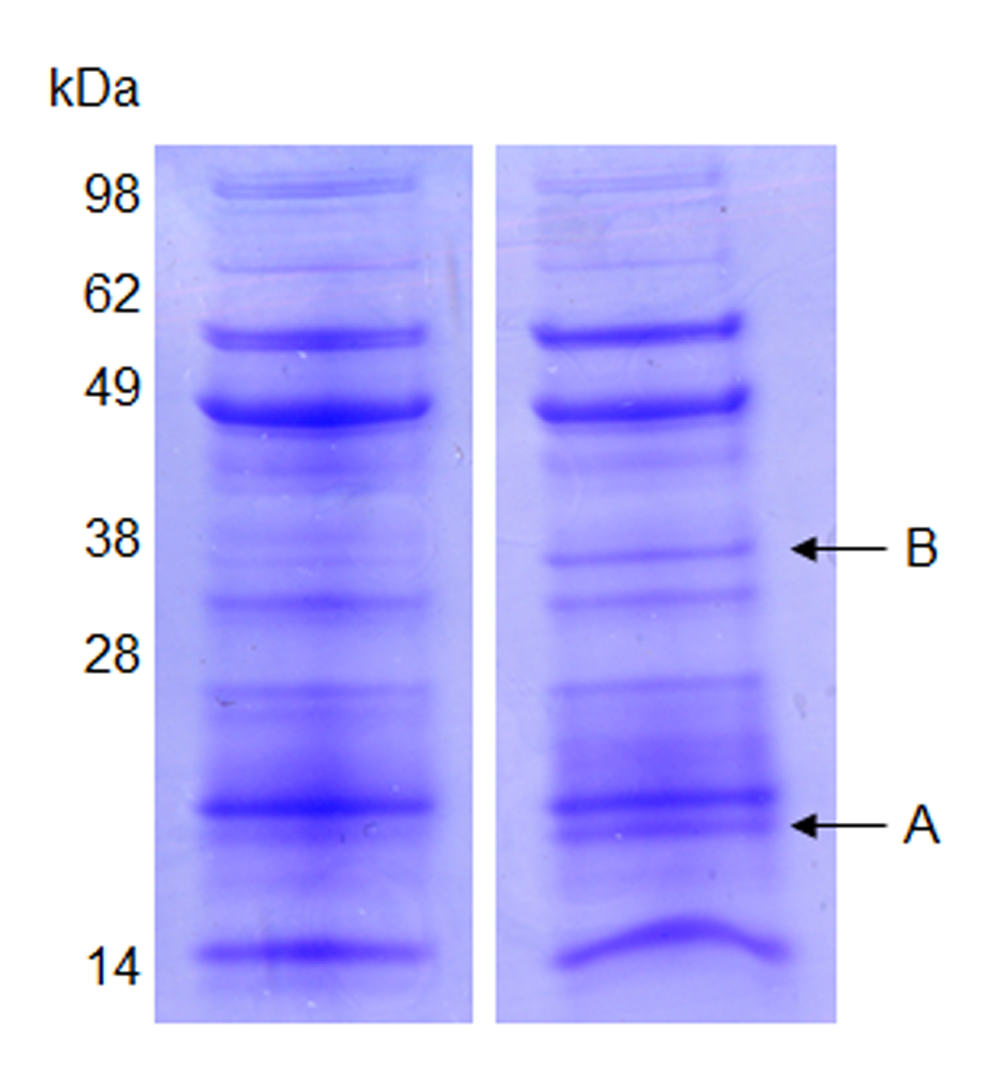

Supplement: Figure S2 — MglA co-purified with hypothetical protein Bd2492 (TPRBd). SDS-PAGE on 10–20% Tris-Tricine gel with protein molecular weights (left), HID13 control (left lane) and HI MglA His8 (right lane). Differential bands are indicated by arrows A and B. Each differential band was excised and analysed by LC-MS/MS. The lower band (A, 22.2 kDa) was identified as Bd3734 (the protein bait) and the upper band (B, 40.5 kDa) was identified as Bd2492. (TIF) [file pgen.1004253.s002.tif]

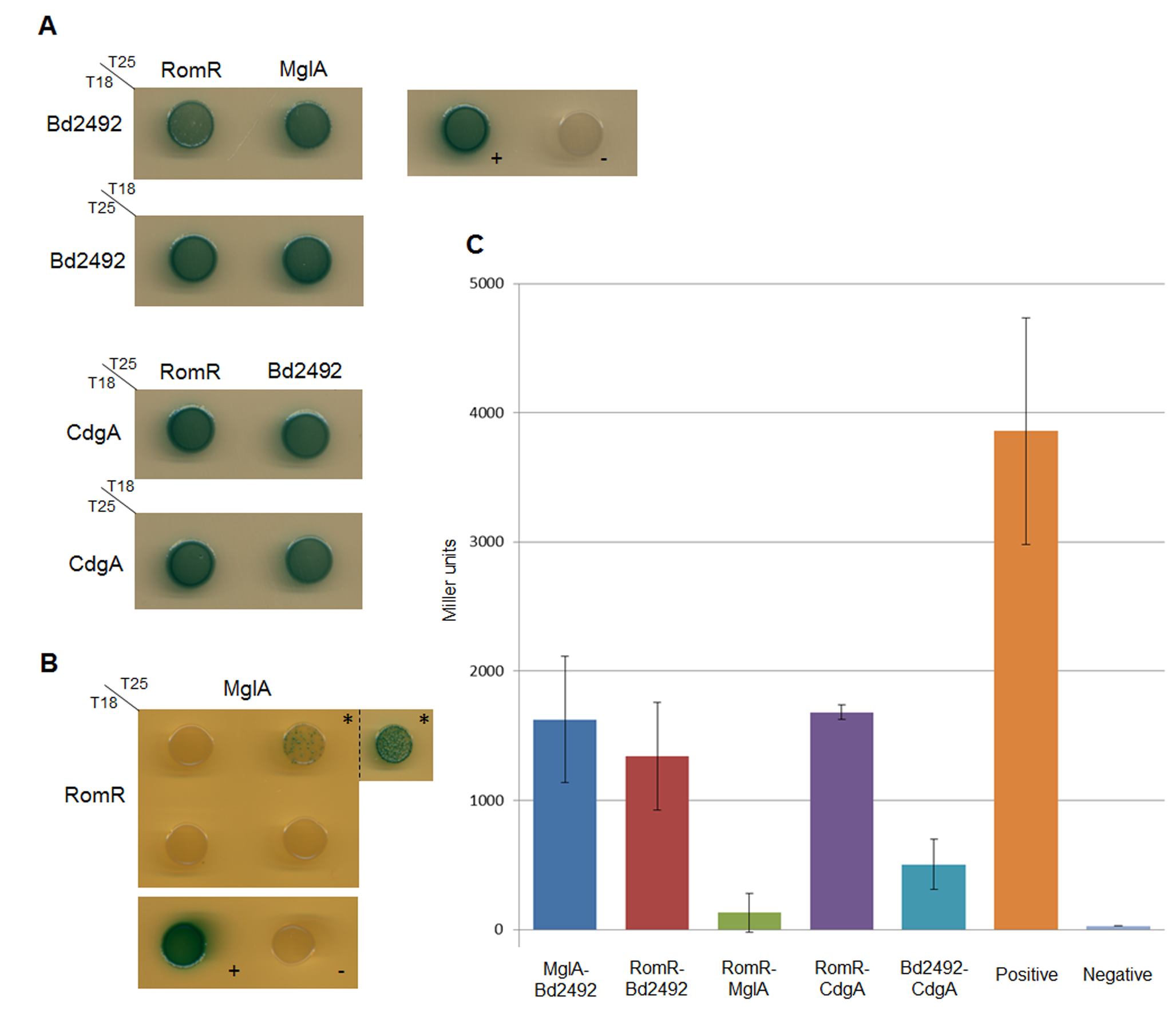

Supplement: Figure S3 — Bacterial two-hybrid shows MglA and RomR interact with Bd2492; RomR and Bd2492 interact with CdgA. A bacterial two-hybrid (BTH) assay between Bd2492 and MglA produces a positive signal on spot tests (A); the interaction between pUT18C-MglA and pKT25-Bd2492 was confirmed by beta-galactosidase assay (C). A positive result was also obtained for a BTH interaction between RomR homologue Bd2761 and Bd2492 on spot tests (A); the interaction between pUT18C-RomR pKT25-Bd2492 was confirmed by beta-galactosidase assay (C). Both RomR and Bd2492 were found to interact with CdgA (Bd3125) by BTH (A). The interactions between pKT25 Bd3125 and pUT18C-RomR or pUT18C-Bd2492 were confirmed by beta-galactosidase assay (C). When MglA and RomR interactions were assayed with tags at either end of the proteins, one combination (pUT18C-RomR and pKNT25-MglA), indicated by an asterisk (2 independent transformants) reproducibly produced a positive result on spot tests suggesting these two proteins interact (B). This interaction could not be confirmed as significant by beta-galactosidase assay, suggesting there is no interaction (as detected by BTH) between RomR and MglA. Positive control (+) = pUT18-zip and pKT25-zip and negative control (−) = pUT18C and pKT25. Error bars represent 1 SD from the mean. (TIF) [file pgen.1004253.s003.tif]

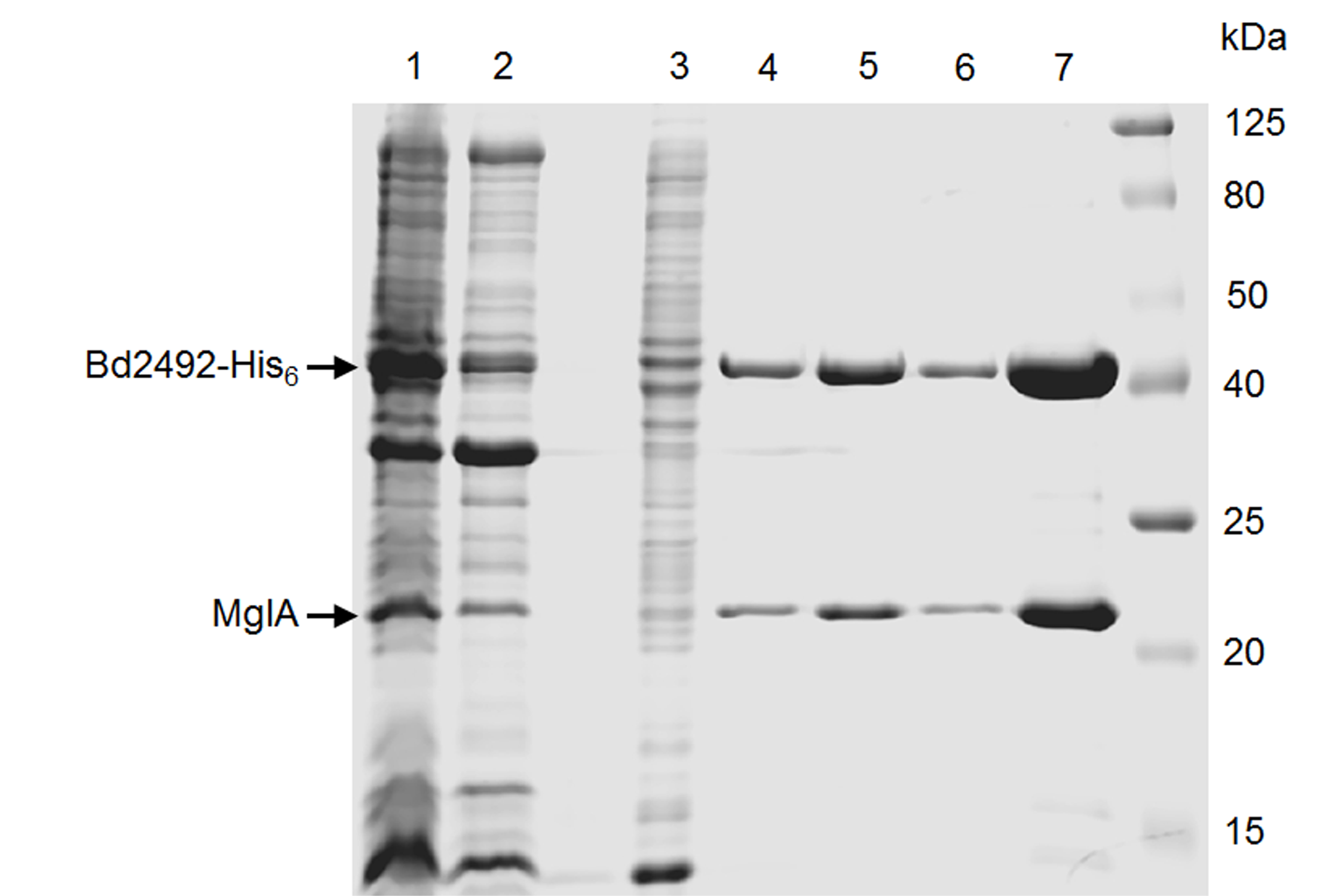

Supplement: Figure S4 — Purification of the MglA-Bd2492-His6 complex. SDS-PAGE of fractions collected during nickel purification of the MglA-Bd2492-His6 complex expressed in E. coli cells harbouring plasmid pD2492N/3734. Soluble E. coli lysate (lane 1); insoluble material (lane 2); flow-through from nickel agarose column (lane 3); proteins eluted from column in the presence of 40 mM imidazole (lanes 4–6) and proteins eluted in the presence of 200 mM imidazole (lane 7). The positions of MglA and Bd2492-His6 on the gel are marked with arrows. (TIF) [file pgen.1004253.s004.tif]

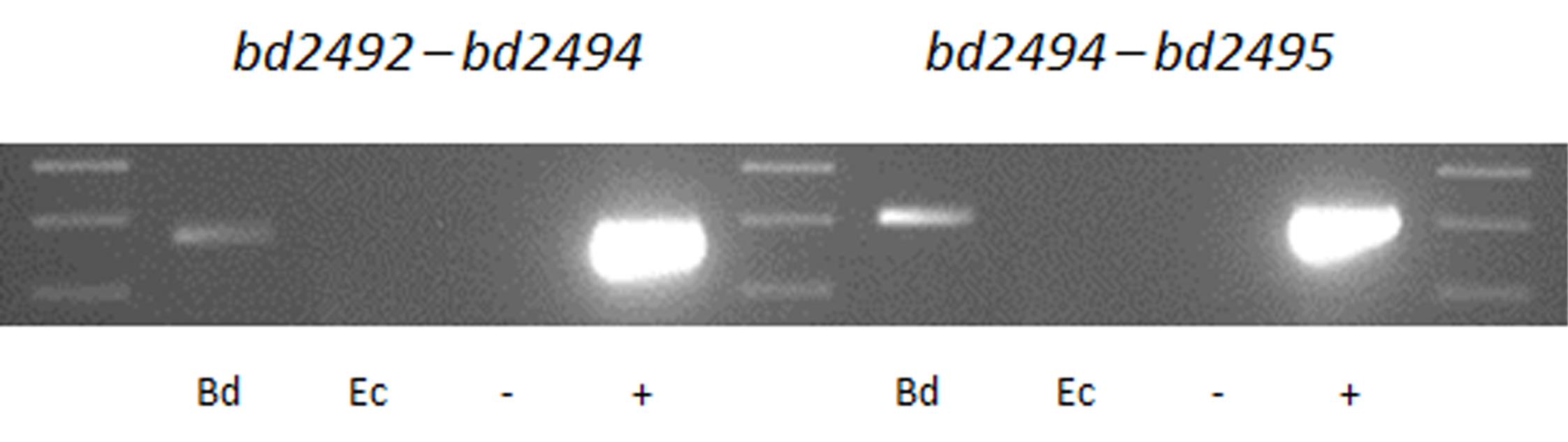

Supplement: Figure S6 — B. bacteriovorus genes bd2492-2495 are co-transcribed. RT-PCR on B. bacteriovorus HD100 attack-phase RNA showed that bd2492 and bd2494 (left) are co-transcribed, as are bd2494 and bd2495 (right). This suggests that the three genes are all co-transcribed in the same operon. Bd = attack-phase B. bacteriovorus RNA; Ec = E.coli S17-1 RNA; (−) no template; (+) B. bacteriovorus genomic DNA. (TIF) [file pgen.1004253.s006.tif]

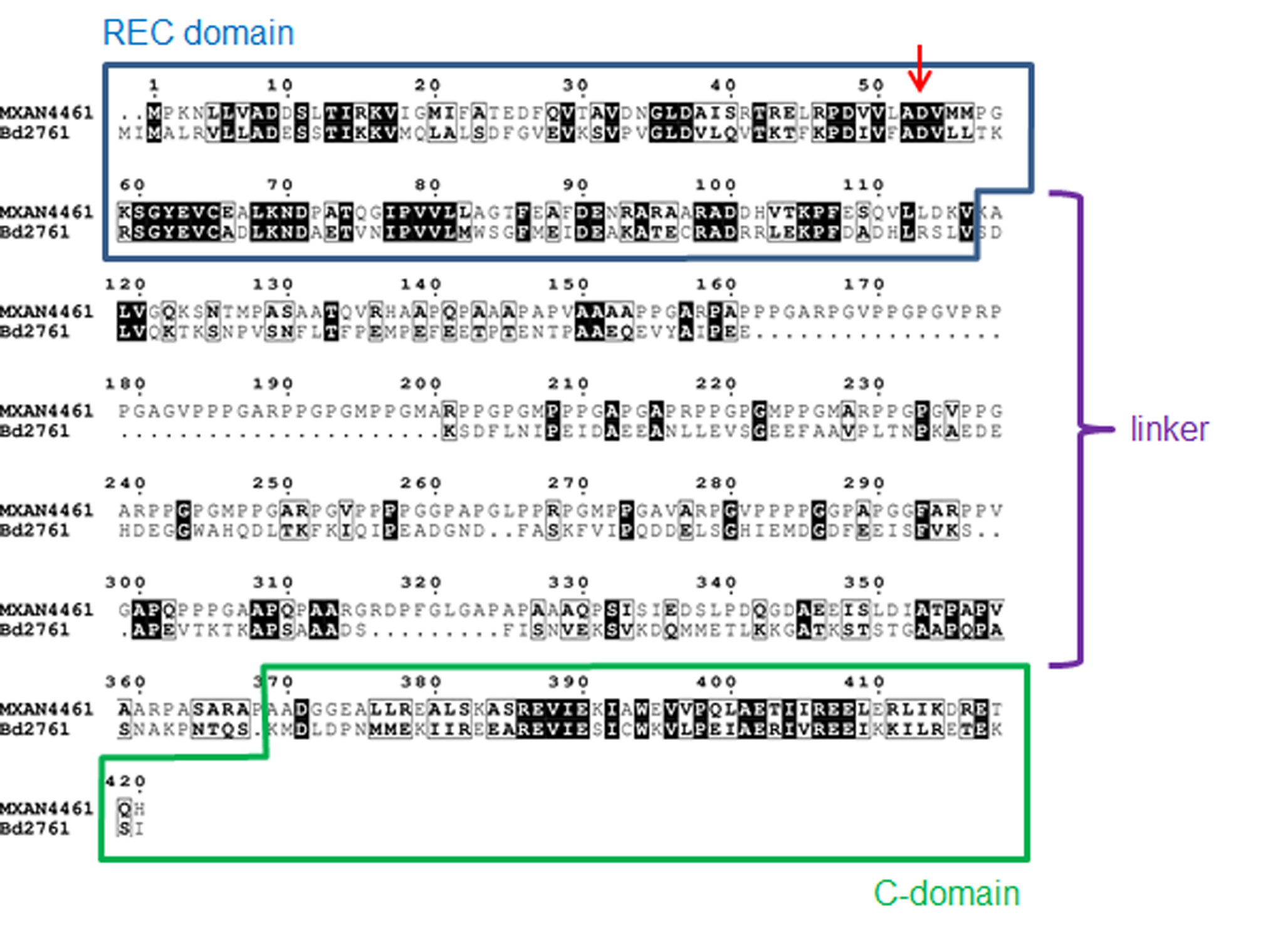

Supplement: Figure S7 — ClustalW protein alignment of M. xanthus RomR (MXAN_4461) and B. bacteriovorus putative RomR homologue Bd2761. The N-terminal REC domain and the C-terminal C-domain are highly conserved between the two proteins, whilst the Pro-rich linker region of M. xanthus RomR (MXAN_4461; accession: YP_632632.1) is not well conserved in Bd2761. A phosphorylatable aspartic acid at residue D53 of M. xanthus (red arrow) is conserved between the two proteins. (TIF) [file pgen.1004253.s007.tif]
