# Supplementary material for: Ras GTPase-Like Protein MglA, a Controller of Bacterial Social-Motility in Myxobacteria, Has Evolved to Control Bacterial Predation by Bdellovibrio
Source: PLoS Genet. 2014 Apr 10;10(4):e1004253. doi: 10.1371/journal.pgen.1004253 (PMC3983030; doi:10.1371/journal.pgen.1004253)
Supplement: Table S1 — Plasmids and strains used in this study. (DOCX) [file pgen.1004253.s008.docx]

**Table S1**

| **Plasmid or strain** | **Description** | **Source** |
| --- | --- | --- |
| ***Plasmids*** | | |
| pK18*mobsacB* | Km^r^ sucrose suicide vector used for conjugation and recombination into *Bdellovibrio* genome | [4] [S7] |
| pK18::∆*mglA* | pK18*mobsacB* containing *mglA* (*bd3734*) deletion and 1Kb flanking DNA | This study |
| pK18::MglA-mCherry | pK18*mobsacB* containing *mglA* (*bd3734*) ORF with C-terminal mCherry tag | This study |
| pK18::MglA-His_8_ | pK18*mobsacB* containing *mglA* (*bd3734*) ORF with C-terminal *catcatcaccatcaccaccatcat* prior to the stop codon. | This study [S5] |
| pK18::*mglA* | pK18*mobsacB* containing *mglA* (*bd3734*) and 199 bp 3’ and 101 bp 5’ flanking DNA for *in cis* complementation | This study |
| pK18::∆*bd2492* | pK18*mobsacB* containing *bd2492* deletion and 1Kb flanking DNA | This study |
| pK18::Bd2492-mCherry | pK18*mobsacB* containing *bd2492* ORF with C-terminal mCherry tag | This study |
| pK18::*bd2492* | pK18*mobsacB* containing *bd2492* and 400 bp 3’ and 113 bp 5’ flanking DNA for *in cis* complementation | This study |
| pK18::Bd2761-mCherry | pK18*mobsacB* containing *bd2761* (*romR*) ORF fragment with C-terminal mCherry tag | This study |
| pZMR100 | λ defective vector, Km^r^. Used to confer Km^r^ for S17-1 used as prey | [4] |
| pMAL-p2_mCherry | Amp^r^ vector containing a *mCherry* gene with a *malE* signal sequence for localised periplasmic fluorescence. | [4] |
| pCL100 | Km^r^ vector encoding *luxCDABE* for luminescence | [1] [S9] |
| pKT25 | Km^r^ vector for fusion of gene to C-terminus of Cya-T25 | [S2] [S11] |
| pUT18C | Amp^r^ vector for fusion of gene to C-terminus of Cya-T18 | [S2] [S10] [S7] |
| pKT25-MglA | pKT25 containing *mglA* (*bd3734*) ORF | This study |
| pKNT25-MglA | pKNT25 containing *mglA* (*bd3734*) ORF | This study |
| pKT25-Bd2492 | pKT25 containing *bd2492* ORF | This study |
| pKT25-RomR | pKT25 containing *bd2761* ORF | This study |
| pKT25-Bd3125 | pKT25 containing *bd3125* ORF | This study |
| pKT25-zip | pKT25 containing the leucine zipper region from yeast GCN4 | [S2] [S11] |
| pUT18C-MglA | pUT18C containing *mglA* (*bd3734*) ORF | This study |
| pUT18C-Bd2492 | pUT18C containing *bd2492* ORF | This study |
| pUT18C-RomR | pUT18C containing *bd2761* ORF | This study |
| pUT18-RomR | pUT18 containing *bd2761* ORF | This study |
| pUT18C-Bd3125 | pUT18C containing *bd3125* ORF | This study |
| pUT18-zip | pUT18C containing the leucine zipper region from yeast GCN4 | [S2] |
| pD2492C/3734 | pCDFDuet-1 (Novagen) with bd2492 and bd3734 coding regions under the control of 2 separate IPTG-inducible T7 promoters | This study |
| ***Escherichia coli*** | | |
| S17-1 | *thi,pro,hsdR*^-^,*hsdM*^+^,*rec*A; integrated plasmid RP4-Tc::Mu-Km::Tn*7*; used as donor for conjugating plasmids into *Bdellovibrio* | [S10] [S8] |
| S17-1::pMAL_p2-mCherry | S17-1 with Amp^r^ plasmid containing *mCherry* gene with a *malE* signal sequence for localised periplasmic fluorescence | [27] |
| BTH101 | F^-^,∆cya99, galE15, galK16, rpsL1, hsdR2, mcrA1, mcrB1 bacterial two-hybrid strain | [2] |
| ***Bdellovibrio bacteriovorus*** | | |
| HD100 | Wild-type *Bdellovibrio* strain, genome sequenced | [S12] |
| HID13 | Host-independent derivative of HD100 | [3] [S13] |
| HID26 | Host-independent derivative of HD100 | [3] |
| HID50 | Host-independent derivative of HD100 | This study |
| HD100 ∆*pilA* HI | Host-independent derivative of HD100 with *pilA* (*bd1290*) markerless deletion | [4] [S14] |
| HD100 ∆*mglA* HI | Host-independent derivative of HD100 with *mglA* (*bd3734*) markerless deletion | This study |
| HD100 ∆*bd2492* HI | Host-independent derivative of HD100 with *bd2492* markerless deletion | This study |
| HD100 ∆*mglA* + pK18 *mglA* | Host-independent derivative of HD100 with *mglA* (*bd3734*) markerless deletion and single-crossover of pK18::*mglA* | This study |
| HD100 MglA-mCherry | HD100 with single-crossover of pK18::MglA-mCherry; *mglA* ORF fused to *mCherry* expressed from native promoter, and promoterless copy of *mglA* | This study |
| HD100 MglA-His_8_ | HD100 with single-crossover of pK18::MglA-His_8_; *mglA* ORF with C-terminal His_8_ expressed from native promoter, and promoterless copy of *mglA* | This study |
| HD100 Bd2761-mCherry | HD100 with single-crossover of pK18::Bd2761-mCherry; *bd2761* ORF fragment fused to *mCherry* expressed from native promoter | This study |
| HD100 Bd2492-mCherry | HD100 with single-crossover of pK18::Bd2492 mCherry; *bd2492* ORF fused to *mCherry* expressed from native promoter, and promoterless copy of *bd2492* | This study |

1. Lambert C, Smith MCM, Sockett RE (2003) A novel assay to monitor predator-prey interactions for *Bdellovibrio bacteriovorus* 109J reveals a role for methyl-accepting chemotaxis proteins in predation. Environ Microbiol 5: 127-132.

2. Karimova G, Dautin N, Ladant D (2005) Interaction network among *Escherichia coli* membrane proteins involved in cell division as revealed by bacterial two-hybrid analysis. J Bacteriol 187: 2233-2243.

3. Lambert C, Ivanov P, Sockett RE (2010) A Transcriptional “Scream” Early Response of *E. coli* Prey to Predatory Invasion by *Bdellovibrio* Curr Microbiol 60: 419-427.

4. Atterbury RJ, Hobley L, Till R, Lambert C, Capeness MJ, et al. (2011) Effects of orally administered *Bdellovibrio bacteriovorus* on the well-being and *Salmonella* colonization of young chicks. Appl Environ Microbiol 77: 5794-5803.
