# Supplementary material for: Ras GTPase-Like Protein MglA, a Controller of Bacterial Social-Motility in Myxobacteria, Has Evolved to Control Bacterial Predation by Bdellovibrio
Source: PLoS Genet. 2014 Apr 10;10(4):e1004253. doi: 10.1371/journal.pgen.1004253 (PMC3983030; doi:10.1371/journal.pgen.1004253)
Supplement: Table S2 — Primers used in this study. (DOCX) [file pgen.1004253.s009.docx]

**Table S2**

| **Primer** | **Sequence (5’ – 3’)** | **Description** |
| --- | --- | --- |
| ***Fluorescent/deletion/complementation constructs*** | | |
| MglA_KO_F1 | TGCGCCGAATTCGAGTACGAAGCAACTTCCGG | Forward primer to amplify approximately 1 kb upstream of *mglA* (*bd3734*), with *Eco*RI site |
| MglA_KO_F2 | GAACATCCAGTGGGTTTACCTCTAGAACGTTCTAAAAGGCGGAACG | Internal primer containing homology to 5’ start and 3’ end of *mglA*, with *Xba*I site |
| MglA_KO_R1 | CGTTCCGCCTTTTAGAACGTTCTAGAGGTAAACCCACTGGATGTTC | Internal primer containing homology to 5’ start and 3’ end of *mglA*, with *Xba*I site |
| MglA_KO_R2 | CGCGCCAAGCTTGCTGGAATGTTTCCAAAGCG | Reverse primer to amplify approximately 1 kb downstream of *mglA* (*bd3734*), with *Hind*III site |
| MglA_comp_F | TCACGAATTCGTCCTGCGATCAAGGAAATC | Forward primer to amplify *mglA* + 199bp 5’ DNA for cloning into pK18*mobsacB* vector, with *Eco*RI site, for complementation |
| mglA_comp_R | CTGCAAGCTTACCTCGGGCCTATTGTAACC | Reverse primer to amplify *mglA* + 101bp 3’ DNA for cloning into pK18*mobsacB* vector, with *Hind*III site, for complementation |
| MglA_tag_F | TCACTGGAATTCATGTCCTTTATTAACTACAATGC | Forward primer to amplify *mglA* with *Eco*RI site |
| MglA_tag_R | GTAACGGGTACCCAGAGTCGTTCCGCCTTTTAG | Reverse primer to amplify *mglA* with *Kpn*I site |
| MglA_His_8__F | TCACTGGAATTCATGTCCTTTATTAACTACAATGC | Forward primer to amplify *mglA* for addition of a polyhistidine tag, with *Eco*RI site |
| MglA_His_8__R | TTAATGATGGTGGTGATGGTGATGATGCAGAGTCGTTCCGCCTTTTAG | Reverse primer to amplify *mglA* with poly(8)histidine tag added prior to stop codon |
| Bd2492_KO_F1 | TCGCCAGGTACCTCCGGTCACCACCAACAAGG | Forward primer to amplify approximately 1 kb upstream of *bd2492* with *Kpn*I site |
| Bd2492_KO_F2 | CTGCGGAGGCTTTTTTCTTGGGATCCTGGGGTGGCCAGTGAGAAGG | Internal primer containing homology to 5’ start and 3’ end of *bd2492*, with *Bam*HI site |
| Bd2492_KO_R1 | CCTTCTCACTGGCCACCCCAGGATCCCAAGAAAAAAGCCTCCGCAG | Internal primer containing homology to 5’ start and 3’ end of *bd2492*, with *Bam*HI site |
| Bd2492_KO_R2 | ACGAACTCTAGATATTCACCCTGAACGCGCGC | Reverse primer to amplify approximately 1 kb downstream of *bd2492* with *Xba*I site |
| Bd2492_comp_F | GCACGAATTCAAACATTCGAACAAATCGCC | Forward primer to amplify *bd2492* + 400bp 5’ DNA for cloning into pK18*mobsacB* vector, with *Eco*RI site, for complementation |
| Bd2492_comp_R | ATGCAAGCTTTTGGATTTTGTTCAGCGCCC | Reverse primer to amplify *bd2492* + 113bp 3’ DNA for cloning into pK18*mobsacB* vector, with *Hind*III site, for complementation |
| Bd2492_tag_F | TCACTGGAATTCTTGTCCACATATATTGAGTTAG | Forward primer to amplify *bd2492* with *Eco*RI site |
| Bd2492_tag_R | GTAACGGGTACCCTGGCCACCCCAGATGCTGAG | Reverse primer to amplify *bd2492* with *Kpn*I site |
| Bd2761_tag_F | GCTCACGAGCTCATGCAACTGGCATTGTCTGA | Forward primer to amplify *bd2761* with *Sac*I site |
| Bd2761_tag_R | CGGAGCGGTACCAATGGACTTTTCAGTTTCGC | Reverse primer to amplify *bd2761* with *Kpn*I site |
| **Bacterial two-hybrid/protein purification** | | |
| MglA_BTH_F | CCAGACTCTAGACATGTCCTTTATTAACTACAA | Forward primer to amplify *mglA* (*bd3734*) with *Xba*I site and additional cytosine |
| MglA_BTH_R | AATGTGGGTACCTTACAGAGTCGTTCCGCCTT | Reverse primer to amplify *mglA* (*bd3734*) with *Kpn*I site for cloning into pUT18C/pKT25 |
| MglA_BTH(C)_R | TATGGATCCGGCAGAGTCGTTCCGCCTTTTAG | Reverse primer to amplify *mglA* (*bd3734*) with *Kpn*I site for cloning into pUT18/pKNT25 |
| Bd2761_BTH_F | CCAGACTCTAGACATGGCTTTACGCGTCTTGCT | Forward primer to amplify *bd2761* (*romR*) with *Xba*I site and additional cytosine |
| Bd2761_BTH_R | AATGTGGGTACCTTAAATGGACTTTTCAGTTTC | Reverse primer to amplify *bd2761* (*romR*) with *Kpn*I site for cloning into pUT18C/pKT25 |
| Bd2761_BTH(C)_R | aatgtgGGATCCGGAATGGACTTTTCAGTTTCGC | Reverse primer to amplify *bd2761* (*romR*) with *Kpn*I site for cloning into pUT18/pKNT25 |
| Bd2492_BTH_F | CCAGACTCTAGACTTGTCCACATATATTGAGTT | Forward primer to amplify *bd2492* with *Xba*I site and additional cytosine |
| Bd2492_BTH_R | AATGTGGGTACCTCACTGGCCACCCCAGATGC | Reverse primer to amplify *bd2492* with *Kpn*I site |
| Bd3125_BTH_F | CCAGACTCTAGACTTGAACATTCGCGATTACAG | Forward primer to amplify *cdgA* (*bd3125*) with *Xba*I site and additional cytosine |
| Bd3125_BTH_R | AATGTGGGTACCCTATTCCGCTGTCACTTCAA | Reverse primer to amplify *cdgA* (*bd3125*) with *Kpn*I site for cloning into pUT18C/pKT25 |
| Bd2492_duet-Fwd | CCATCACCATCATCACCACAGCCAGATGTCCACATATATTGAGTTAGAAATCC | Forward primer to amplify *bd2492* for restriction-free cloning into pCDFDuet-1 |
| Bd2492_duet_Rev | CGATTACTTTCGTTCGACTTAAGCATTACTGGCCACCCCAGATGCTGAGC | Reverse primer to amplify *bd2492* for restriction-free cloning into pCDFDuet-1 |
| Bd3734_duet_Fwd | GTTAAGTATAAGAAGGAGATATACATATGTCCTTTATTAACTACAATGCC | Forward primer to amplify *bd3734* for restriction-free cloning into pCDFDuet-1 |
| Bd3734_duet_Rev | GGTGGCAGCAGCCTAGGTTAATTACAGAGTCGTTCCGCCTTTTAG | Reverse primer to amplify *bd3734* for restriction-free cloning into pCDFDuet-1 |
