# Supplementary material for: Ras GTPase-Like Protein MglA, a Controller of Bacterial Social-Motility in Myxobacteria, Has Evolved to Control Bacterial Predation by Bdellovibrio
Source: PLoS Genet. 2014 Apr 10;10(4):e1004253. doi: 10.1371/journal.pgen.1004253 (PMC3983030; doi:10.1371/journal.pgen.1004253)
Supplement: Text S1 — Supplemental Materials and Methods. (DOCX) [file pgen.1004253.s010.docx]

**Text S1: Supplemental Materials and Methods**

**Construction of *B. bacteriovorus* deletion mutants**

To construct *mglA* (*bd3734*) and *bd2492* deletion strains, the following strategy was employed: approximately 1 Kb regions upstream and downstream of the gene to be deleted were amplified and joined together using Phusion polymerase (NEB). The *bd2492* deletion construct retained the first 3 bp and the last 15 bp of the gene. In order to avoid confounding consequences of their removal, the *mglA* construct retained the first 100 bp of *mglA*, likely containing a *recR* terminator region, and the last 29 bp of the gene, so as to include predicted -10 and -35 consensus sequences of the promoter of downstream *nadA*. The deletion constructs were designed in plasmid pK18*mobsacB* and transformed into the *E. coli* donor strain S17-1. The plasmid was then conjugated into *Bdellovibrio* *bacteriovorus* HD100, and kanamycin-resistant merodiploid *Bdellovibrio* strains were selected by growth on S17-1 pZMR100 lawns on YPSC overlay plates supplemented with 50 µg ml^-1^ kanamycin.

The host-dependent merodiploid strains, containing a single crossover from the plasmid to the genome, were grown in the presence of sucrose (pK18*mobsacB* is a sucrose suicide vector) to induce a second recombination event, and plated on S17-1 lawns on YPSC overlay plates. Kanamycin-sensitive *Bdellovibrio* strains were screened for the required deletion.

For both *mglA* and *bd2492*, a host-dependent deletion strain could not be obtained; however HI isolates of the merodiploid strains were created as described previously [4] and plated onto PY agar [S1] without selection. Kanamycin-sensitive HI *Bdellovibrio* strains were assessed for the required deletion by colony PCR. The markerless deletion HI strains for ∆*mglA* and ∆*bd2492* were then confirmed by PCR and Southern blotting/reverse-transcriptase (RT) PCR.

***mglA* and *bd2492* complementation**

Complementation of the ∆*mglA* strain was achieved, *in cis*, using pK18*mobsacB* carrying the *mglA* ORF plus 199 bp of 5’ DNA and 101 bp 3’ DNA: a single-crossover method described previously [25]. The *mglA* ORF and flanking region was amplified using primers MglA_comp_F and MglA_comp_R. The resulting plasmid was conjugated into the HD100 ∆*mglA* HI strain, and single-crossover exconjugants we selected by growth on PY agar supplemented with kanamycin at 50 µg ml^-1^. Complementation of the ∆*bd2492* strain was achieved using the same strategy (using primers Bd2492_comp_F and Bd2492_comp_R to amplify the *bd2492* ORF plus 400 bp 5’ and 113 bp 3’ DNA). Exconjugants were tested for predation.

**Tagging and *Bdellovibrio-*expression of proteins with C-terminal mCherry**

The ORF for each gene was amplified from *B. bacteriovorus* HD100 genomic DNA using Phusion polymerase (NEB) with a primer to the start of the gene and a primer which replaced the stop codon with a KpnI site. These were then fused in-frame with the cognate gene (*mglA_Bd_, romR_Bd_* or bd2492*)* encoding mCherry fluorescent protein to generate a protein with an in-frame C-terminal monomeric mCherry protein via a short peptide linker of amino acids GTSS, as described previously [25] . The whole gene ORF was used to create a merodiploid strain with both a promoterless wild-type and a tagged copy of the gene using the native promoter. A similar process was used to create *mglA_Bd_-*His_8_ except the reverse primer introduced the DNA encoding octa-histidine.

The gene fused to the *mCherry* gene, or *His_8_*, was then cloned into plasmid pK18*mobsacB* and conjugated into *B. bacteriovorus* HD100 as described above. Single-crossover exconjugants were selected for, and maintained, by growth in media supplemented with kanamycin at 50 µg ml^-1^.

**Bacterial two hybrid (BTH) assay**

For bacterial two hybrid analysis, each ORF was cloned in-frame with the T18 and T25 fragments of adenylate cyclase in vectors pUT18/pUT18C and pKNT25/pKT25 [S2].The resulting vectors were then co-transformed into *E. coli* strain BTH101 and plated onto Nutrient Agar (Oxoid) supplemented with 50 µg ml^-1^ Ampicillin, 25 µg ml^-1^ Kanamycin and 40 µg ml^-1^ 5-bromo-4-chloro-3-indolyl-β-D-galactopyranoside (X-gal) and incubated at 29°C for 48 hours. Three transformants for each assay were cultured to stationary phase in LB broth and spotted onto nutrient agar, supplemented as above and incubated for 48 hours at 29°C. Plates were then scanned on an Epson Perfection 1200U scanner. Beta-galactosidase activity was performed at 29°C on 1 ml stationary-phase aliquots of cultures as described by Miller [S3].

**Host-independent attachment assay**

An assay was used to assess the ability of different engineered *Bdellovibrio* HI strains to attach to *E. coli* prey cells. Briefly, 2.5 ml HI culture (grown in PY to OD_600_ 1.5-2.0) was re-suspended in 1 ml CaHEPES, and 10 ml *E. coli* S17-1 (grown in YT broth for 16 hours at 37°C 200 rpm) was back-diluted to OD_600_ 1.0 in CaHEPES. These cultures were incubated at 29°C for 30 minutes to allow the cells to recover. The infection was started by addition of 1 ml HI *B. bacteriovorus* culture, 800 µl *E. coli* S17-1 and 600 µl CaHEPES to a universal tube, and this was incubated at 29°C for 1 hour. Attachment percentages were then analysed by microscopy of samples immobilized on 1% agarose/CaHEPES.

**Host-independent invasion assay**

To assay whether engineered HI strains could invade *E. coli* and form bdelloplasts, the fluorescent *E.coli* S17-1::pMAL_p2-mCherry prey strain [27] was used. 200 µl *B. bacteriovorus* HI strain (grown in PY to OD_600_ 1.5-2.0) and 200 µl *E.coli* S17-1::pMAL_p2-mCherry (from a culture grown for 16 hours at 37°C 200 rpm in YT broth supplemented with Amp^50^ and IPTG^200^) were re-suspended together in 80 µl CaHEPES and incubated at 29°C for 22 hours. The mixtures were then assayed for bdelloplasts using fluorescent microscopy; the fluorescent prey ‘backlit’ the *B. bacteriovorus* cells inside, allowing for easy identification of bdelloplasts.

**Host-independent predation assay**

To test the completion of predation from attachment and invasion to killing and prey exit, the predation efficiency of HI mutant strains was assayed against *Bdellovibrio* wild-type HI isolates HID13, HID26 and a wild type revertant HI strain (HID50) isolated during screening for an *mglA* deletion strain. HI strains were matched (compensating for diverse cell morphologies) by protein concentration [4] [26] using a Lowry protein assay [S4] to a cell suspension equivalent to 500 µg total protein, and this was added to 10 ml *E. coli* S17-1 (grown in YT broth for 16 hours at 37°C 200 rpm ) diluted to OD_600_ 1.0 in CaHEPES. *E. coli* numbers were enumerated on YT agar at 24 hour intervals, for 48 hours.

**Co-purification assay**

Protein co-purification assays used a modified technique [S5] of the Mignot lab [24]. HI *Bdellovibrio* strains were grown to an OD_600_ of 0.6 in PY Km^50^ (HID13 control was grown in PY) at 29°C, 200 rpm. The cells were collected by centrifugation at 5,000 rpm, and the supernatant discarded. The pellet was washed twice in wash buffer (NaH_2_PO_4_ 50 mM, NaCl 300 mM, MgCl_2_ 5 mM, pH 8.0) and resuspended in 20ml lysis buffer (NaH_2_PO_4_ 50 mM, NaCl 300 mM, MgCl_2_ 5 mM, 3 µl β-mercaptoethanol, 200 µl Protease Inhibitor Cocktail (Thermo Scientific), 3 µl DNaseI, 20 mM Imidazole pH 8.0). The cells were disrupted by sonication and centrifuged at 18,000 rpm 4°C for 1 hour. The supernatant was transferred to a 50 ml Falcon tube, mixed with pre-equilibrated TALONspin^TM^ Cobalt beads and incubated at 4°C for 2 hours on a rocker. The bead-bound MglA complexes were collected by centrifugation at 2,500 rpm and washed twice in wash buffer. The bead-bound complexes were eluted by addition of 100 µl protein loading buffer directly to the beads, and boiling 100°C for 10 minutes.

The samples were then run on a 10-20% Tris-tricine SDS PAGE gel (BioRad), stained using Coomassie Blue stain and destained with destain solution (methanol:acetic acid:distilled water; 3:1:6). Bands were excised from the gel, tryptically digested and subjected to LC-MS/MS analysis using standard techniques as described previously [1].

**Purification of heterologously expressed Bd2492 and MglA from *E. coli***

The coding regions of Bd2492 and Bd3734 were amplified by PCR using primers Bd2492_duet-Fwd/Rev and Bd3734_duet_Fwd/Rev, respectively. The resulting PCR fragments were introduced to plasmid pCDFDuet-1 (Novagen) by two successive rounds of restriction-free cloning as described by van den Ent and Lowe [S6].The resulting plasmid, pD2492C/3734, includes the coding regions of the two *Bdellovibrio*-derived genes under the control of two separate IPTG-inducible T7 promoters with Bd2492 being fused in-frame to an N-terminal His_6_-tag.

Cultures of *E. coli* T7 Express cells (New England Biolabs) harbouring plasmid pD2492C/3734 were grown at 37°C to an OD_650_ of 0.8 and induced with 0.5 mM IPTG. Cells were grown for a further 20 hours at 20˚C before harvesting by centrifugation. All purification steps were performed at 4°C. Cell pellets were re-suspended in Buffer A (20 mM imidazole pH 7.0, 400 mM NaCl, 5% glycerol, 0.05% Tween20) supplemented with 1 mg/ml lysozyme and then lysed via sonication. The resulting lysate was clarified by centrifugation and used in nickel-affinity chromatography with elution of bound proteins being achieved with the addition of Buffer A supplemented with 400 mM imidazole.

For analytical gel filtration experiments, fractions of nickel-affinity purified proteins were concentrated by centrifugation on a 10 KDa MWCO Vivaspin column (Sartorius) and then separated on a Superdex 26/60 200 column (GE Healthcare) pre-equilibrated with Buffer B (ADA NaOH pH 6.0, 200 mM NaCl, 2 mM β-mercaptoethanol).

**Supplemental references:**

S1. Shilo M, Bruff B (1965) Lysis of gram-negative bacteria by host-independent ectoparasitic *Bdellovibrio bacteriovorus* isolates. J Gen Microbiol 40: 317-328.

S2. Karimova G, Ullmann A, Ladant D (2001) Protein-protein interaction between *Bacillus stearothermophilus* tyrosyl-tRNA synthetase subdomains revealed by a bacterial two-hybrid system. J Mol Microbiol Biotechnol 3: 73-82.

S3 Miller JH (1972) Experiments in molecular genetics. Cold Spring Harbor, N.Y.: Cold Spring Harbor Laboratory.

S4. Lowry OH, Rosebrough NJ, Farr AL, Randall RJ (1951) Protein measurement with the Folin phenol reagent. J Biol Chem 193: 265-275.

S5. Allers T, Barak S, Liddell S, Wardell K, Mevarech M (2010) Improved Strains and Plasmid Vectors for Conditional Overexpression of His-Tagged Proteins in *Haloferax volcanii*. Appl Environ Microb 76: 1759-1769.

S6. van den Ent F, Lowe J (2006) RF cloning: A restriction-free method for inserting target genes into plasmids. J Biochem Biophys Methods 67: 67-74.

S7. Schafer A, Tauch A, Jager W, Kalinowski J, Thierbach G, et al. (1994) Small mobilizable multi-purpose cloning vectors derived from the *Escherichia coli* plasmids pK18 and pK19: selection of defined deletions in the chromosome of *Corynebacterium glutamicum*. Gene 145: 69-73.

S8 Rogers M, Ekaterinaki N, Nimmo E, Sherratt D (1986) Analysis of Tn7 transposition. Mol Gen Genet 205: 550-556.

S9. Lambert C, Smith MCM, Sockett RE (2003) A novel assay to monitor predator-prey interactions for *Bdellovibrio bacteriovorus* 109J reveals a role for methyl-accepting chemotaxis proteins in predation. Environ Microbiol 5: 127-132.

S10. Simon R, Preifer U, Puhler A (1983) A broad host range mobilisation system for *in vivo* genetic engineering: transposon mutagenesis in gram negative bacteria. Biotechnology 9: 184-191.

S11. Karimova G, Dautin N, Ladant D (2005) Interaction network among *Escherichia coli* membrane proteins involved in cell division as revealed by bacterial two-hybrid analysis. J Bacteriol 187: 2233-2243.

S12. Rendulic S, Jagtap P, Rosinus A, Eppinger M, Baar C, et al. (2004) A predator unmasked: life cycle of *Bdellovibrio bacteriovorus* from a genomic perspective. Science 303: 689-692.

S13. Lambert C, Ivanov P, Sockett RE (2010) A Transcriptional “Scream” Early Response of *E. coli* Prey to Predatory Invasion by *Bdellovibrio* Curr Microbiol 60: 419-427.

S14. Atterbury RJ, Hobley L, Till R, Lambert C, Capeness MJ, et al. (2011) Effects of orally administered *Bdellovibrio bacteriovorus* on the well-being and *Salmonella* colonization of young chicks. Appl Environ Microbiol 77: 5794-5803.
